# Coupled hard–soft spinel ferrite-based core–shell nanoarchitectures: magnetic properties and heating abilities†

**DOI:** 10.1039/d0na00134a

**Published:** 2020-05-06

**Authors:** Marco Sanna Angotzi, Valentina Mameli, Claudio Cara, Anna Musinu, Claudio Sangregorio, Daniel Niznansky, Huolin L. Xin, Jana Vejpravova, Carla Cannas

**Affiliations:** Department of Chemical and Geological Sciences, University of Cagliari S.S. 554 bivio per Sestu 09042 Monserrato (CA) Italy ccannas@unica.it; Consorzio Interuniversitario Nazionale per la Scienza e Tecnologia dei Materiali (INSTM) Via Giuseppe Giusti 9 50121 Firenze (FI) Italy; Istituto di Chimica dei Composti OrganoMetallici - Consiglio Nazionale delle Ricerche (ICCOM-CNR) Via Madonna del Piano 10 50019 Sesto Fiorentino (FI) Italy; Department of Chemistry “U. Schiff”, University of Florence Via della Lastruccia 3-13 50019, Sesto Fiorentino (FI) Italy; Department of Inorganic Chemistry, Charles University Hlavova 8 12800 Prague 2 Czech Republic jana@mag.mff.cuni.cz; Center for Functional Nanomaterials, Brookhaven National Laboratory 735 Brookhaven Ave Upton NY 11973 USA; Department of Physics and Astronomy, University of California Irvine CA 92697 USA; Department of Condensed Matter Physics, Charles University Ke Karlovu 5 12116 Prague 2 Czech Republic; Consorzio per la Promozione di Attività Universitarie Sulcis-Iglesiente (AUSI), Centro di Ricerca per l'Energia, l'Ambiente e il TErritorio (CREATE) Palazzo Bellavista Monteponi 09016 Iglesias (CI) Italy

## Abstract

Bi-magnetic core–shell spinel ferrite-based nanoparticles with different CoFe_2_O_4_ core size, chemical nature of the shell (MnFe_2_O_4_ and spinel iron oxide), and shell thickness were prepared using an efficient solvothermal approach to exploit the magnetic coupling between a hard and a soft ferrimagnetic phase for magnetic heat induction. The magnetic behavior, together with morphology, stoichiometry, cation distribution, and spin canting, were investigated to identify the key parameters affecting the heat release. General trends in the heating abilities, as a function of the core size, the nature and the thickness of the shell, were hypothesized based on this systematic fundamental study and confirmed by experiments conducted on the water-based ferrofluids.

## Abstract 2

The heating abilities of bi-magnetic core–shell nanostructures are studied depending on core size, nature of the shell, and shell thickness.

## Graphical abstract

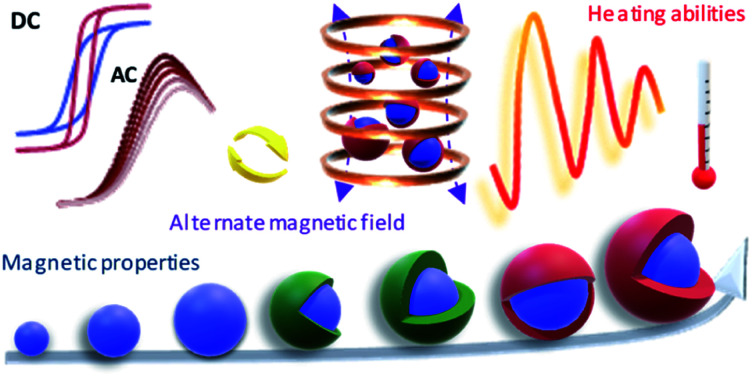

## Introduction

Coupled bi-magnetic nanoparticles (NPs) exhibit outstanding properties, strongly dependent on the extent and nature of the interface.^1–4^ Antiferromagnet (AFM)/ferromagnet (FM) and AFM/ferrimagnet (FiM) interfaces are the most frequently studied systems. Their magnetic behaviour is characterized by exchange bias, which is manifested by an increase and asymmetry in the coercivity, giving rise to a shift of the hysteresis loop along the magnetic field axis.^5–9^ Since the pioneering work by Kneller and Hawig,^10^ many studies have focused on the investigation at the nanoscale of the coupling phenomena between hard and soft FM or FiM phases.^11^ In particular, the magnetic behaviour of thin films has been deeply studied and described as a specific superposition of the intrinsic parameters of the *hard* and *soft* phases depending on the coupling strength between the phases. Indeed, *hard* phases feature large magnetic anisotropy (expressed in terms of anisotropy constant, *K*) and moderate saturation magnetization (*M*_s_), while *soft* phases present low *K* and large *M*_s_. In thin films, the magnetization switching behaviour (*i.e.*, the hysteresis loop) depends on the relative dimension (thickness of the layer) of the *hard* and *soft* phases. When the *soft* phase thickness is lower than the double of the domain wall thickness of the *hard* one (*t*_s_ ≪ 2 *δ*_h_), the phases are rigidly coupled and result in a rectangular stage-loop since the magnetization is reversed at the same nucleation field (*H*_N_). In the case of thicker *soft* phases, the system behaves like a “spring-magnet,” showing different switching of the magnetization and, therefore a two-step loop.^11^ Strongly coupled *hard–soft* heterostructures are desired for several applications. For example, they can be employed to replace rare-earth-based magnets, ideally combining the attractive properties of the *hard* (large *K*) and *soft* (high *M*_s_) phases.^12–16^ The thermal stability of the *hard* component, combined with the ability of the *soft* part to reduce the switching field may be exploited in recording media, where to increase the areal bit density it is necessary to avoid the superparamagnetic limit without having such extensive magnetic anisotropy that would prevent the write-head to write the information.^15,17,18^ Again, the possibility to tune magnetic anisotropy and saturation magnetization has recently found usage in applications based on magnetic heat generation, such as catalysis^19–22^ or magnetic fluid hyperthermia (MFH).^23–38^ In this context, the engineering of bi-magnetic core–shell nanoarchitectures with sharp interfaces, homogeneous coating, and low size dispersity for a uniform magnetic response is crucial for maximizing the coupling between the *hard* and *soft* phases (*i.e.*, the interface) and requests proper synthesis methods able to guarantee a strict control over the composition, structure, and morphology of the particles.^39–43^ Isostructural phases with similar cell parameters should ensure epitaxial growth and can be considered the best candidates to build core–shell architectures. From this point of view, spinel ferrites are particularly appealing as they offer a broad spectrum of *hard* and *soft* magnetic behaviours, all sharing the same crystal structure. However, a systematic study of the magnetic parameters influencing the coupling, as already performed for thin films,^44^ is still lacking for core–shell NPs. Some studies^23–38^ have reported the heating abilities of these systems since Lee *et al.* work in 2011 (Table 7S†).^33^ Nevertheless, only a few of them discuss the data based on a large set of samples,^28,31,35–37^ and rarely the variation of a single parameter is studied keeping the other unchanged.^23,24,30,31,33,35,37,38^ More importantly, the undoubted demonstration of the core–shell architecture by nanoscale chemical mapping,^26,32,33^ and the actual chemical composition^23,28,32,35,38^ are hardly ever provided. All the above-cited aspects are essential as a solid starting point to ascribe the increased efficiency of systems to the exchange-coupled spinel ferrite phases. Generally, the heat release efficiency is explained in terms of optimized size, saturation magnetization, and effective magnetic anisotropy caused by exchange interaction. Nonetheless, some other aspects are usually ignored, as the cation distribution and the spin canting, even though their influence on the magnetic properties has been largely demonstrated.^45–48^ Notwithstanding important results that have been achieved on these promising systems in terms of heat release, the available literature data are still limited, and systematic studies are necessary to better understand the correlation between materials' feature and heat dissipation mechanism.

Recently, we set up an alternative synthesis method able to produce homogeneous core–shell NPs with high crystallinity, low dispersity, and precise control of the shell growth, as proven by a detailed characterization by XRD, STEM-EELS, STEM-EDX, FTIR, TGA, and room temperature ^57^Fe Mössbauer spectroscopy.^43^

Here we propose a systematic study of the DC/AC magnetic properties of three *hard* cobalt ferrite (CoFe_2_O_4_) cores covered with *soft* spinel iron oxide (magnetite Fe_3_O_4_/maghemite γ-Fe_2_O_3_) and manganese ferrite (MnFe_2_O_4_), characterized for their actual chemical composition (ICP-AES), their nanoscale architecture and morphology (STEM-EDX), cation distribution, and spin canting (low-temperature ^57^Fe Mössbauer spectroscopy). The evaluation of magnetic parameters provides hypotheses of general trends in the heating abilities as a function of the core size, the nature and the thickness of the shell, that have been then compared with experimental heat abilities obtained from aqueous ferrofluids.

## Experimental

### Methods

Three samples of CoFe_2_O_4_ NPs of different sizes, labelled as CoA, CoB, and CoC, were prepared by the solvothermal hydrolysis of mixed cobalt-iron oleates in a mixture of organic solvents with different polarities and water contents. The samples were used as seeds to produce core–shell nanostructures using a second solvothermal treatment (seed-mediated growth), with a shell of spinel iron oxide (maghemite/magnetite) and manganese ferrite, indicated as Cox@Fe and Cox@Mn (where x = A, B, C), respectively.^43^ The samples CoA, CoA@Fe, CoA@Mn, CoC, CoC@Fe, and CoC@Mn correspond to the samples Co1, Co1@Fe, Co1@Mn, Co2, Co2@Fe, and Co2@Mn, respectively, described in previous work.^43^ The experimental synthesis conditions for the samples CoB, CoB@Fe, and CoB@Mn are reported in the section “*Experimental conditions*” of the ESI (Tables 8S–10S†).

### Equipment

The samples were characterized by X-ray diffraction (XRD), using a PANalytical X'Pert PRO with Cu Kα radiation (1.5418 Å), a secondary monochromator, and a PIXcel position-sensitive detector. The peak position and instrumental width were calibrated using powder LaB_6_ from NIST. The hexane dispersions were dried on a glass plate and measured in the angular range 10–90° with step 0.039°. The experimental diffraction patterns were fitted using the FullProf program.^49^ The microstructural effects were treated using the Voigt approximation as implemented in the code: both instrumental and sample intrinsic profiles are supposed to be described by a convolution of Lorentzian and Gaussian components. The Gaussian and Lorentzian components of the instrumental function were determined using the diffraction pattern of the LaB_6_ standard recorded for the identical instrumental set up, and the sample broadening was approximated by the Gaussian component only. In the structural model, the effects of spinel inversion and the core–shell nature approximated as a two-phase model were tested.

Transmission electron microscopy (TEM) images were obtained using a JEOL 200CX operating at 160 kV. The particle size distribution was obtained by measuring in the automatic mode over 1000 particles through the software Pebbles and adopting a spherical shape.^50^ The mean particle diameter was calculated as the average value and the dispersity as the percentage ratio between the standard deviation and the average value.

High resolution TEM (HRTEM) images and EDX measurements were carried out in the STEM mode using an FEI Talos F200X with a field-emission gun operating at 200 kV equipped with a four-quadrant 0.9-sr energy dispersive X-ray spectrometer. The single line profiles were calculated using the Matlab command “improfile” for different sections all over a particle (*i.e.* over 360° with a step of 0.1°), and the results averaged.

^57^Fe Mössbauer spectroscopy was done on a Wissel spectrometer using transmission arrangement and proportional detector LND-45431. An α-Fe foil was used as a standard, and the fitting procedure was done by the NORMOS program. The in-field measurements were done in a perpendicular arrangement of the external magnetic field with respect to the γ-beam and were used to get information about the cationic distribution and the canting phenomena in the spinel structure (see paragraph “*Low-temperature Mössbauer spectroscopy*” in the ESI, eqn (1S)†).

The chemical composition was studied by inductively coupled plasma-atomic emission spectroscopy (ICP-AES). The dried samples were digested using HNO_3_. The digested sample solutions were stirred at room temperature for 1 h, then heated up to ∼50 °C for 2 h. The solutions were allowed to cool down, filtered, and diluted using 1% v/v HNO_3_ solution. The ICP-AES measurements were performed on a Liberty 200 ICP Varian spectrometer under the following conditions: Fe line: 259.940 nm, Co line: 238.892 nm, Mn line: 257.610 nm; Fe, Co, and Mn concentration range: (0.1/1.5) ppm; Fe detection range: (0.015/750) ppm, Co detection range: (0.050/2500) ppm, Mn detection range: (0.003/150) ppm. The analyses were performed twice on different portions of the samples. The chemical formulas were calculated by assuming the absence of anion vacancies.

The magnetic property measurements were carried out using a SQUID magnetometer (MPMS7XL, Quantum Design). The temperature dependencies of magnetization in the zero field cooled (ZFC) and field cooled (FC) regimes were measured as follows: first, the sample was cooled down to 10 K in the zero external magnetic field. Next, the field of 10 mT was applied, and the temperature dependence of magnetization was measured on heating. Afterward, the sample was cooled down in the applied field of 10 mT, and the temperature dependence of magnetization was measured again. The magnetization isotherms were recorded up to 7 T at selected temperatures in both polarities of the applied magnetic field. All data were corrected according to the organic content. AC susceptibility measurements were recorded with the amplitude of 0.3 mT and in the frequency range of 0.1–1000 Hz between temperatures of 10–400 K. The approach for the evaluation of magnetic parameters, including exact formulas, is reported in the ESI† (see paragraphs “*DC Magnetometry*”, “*AC Magnetometry on powdered samples*”, and “*AC/DC Magnetometry on hydrophilic ferrofluids*”).

Calorimetric estimation of specific absorption rate (SAR) was carried out using a non-adiabatic experimental set-up built at the LAboratorio di Magnetismo Molecolare (LA.M.M.) using a power supply CELESs MP6/400 (FIVES CELES), a water-cooled heating station connected to the power supply, and an induction coil. Heating curves were recorded under a magnetic field of 17 kA m^−1^ and 183 kHz for 300 s on water colloidal dispersions of the magnetic NPs. The hydrophobic NPs were made hydrophilic by an intercalation process with cetyltrimethylammonium bromide (CTAB, (C_16_H_33_)N(CH_3_)_3_Br).^45^ The concentration of the colloidal dispersion was 3.4 mg mL^−1^ for all samples. The temperature of the sample was monitored by an optical fiber probe (OPTOCON-FOTEMP) dipped into the solution. The sample holder was surrounded by polystyrene and hosted in a glass Dewar, equipped by an ethylene glycol thermostat, to ensure the proper thermal insulation. The SAR, *i.e.*, the thermal power per mass unit, values have been estimated by a linear curve fitting in the first 20 s of the heating curves (initial slope method).

## Results and discussion

Three samples of CoFe_2_O_4_ NPs of different sizes (CoA, CoB, and CoC) and their corresponding core–shell nanostructures (Cox@Mn and Cox@Fe, where Mn = MnFe_2_O_4_ and Fe = γ-Fe_2_O_3_/Fe_3_O_4_; x = A, B, C) were synthesized by solvothermal hydrolysis of metal-oleates in a mixture of organic solvents and water.^43,51–53^ Two different shell thicknesses, termed as CoC@Mn1/CoC@Mn2 and CoC@Fe1/CoC@Fe2, were prepared for CoC-based core–shell samples. The samples CoA, CoC, and respective core–shell NPs (except CoC@Mn1 and CoC@Fe1) have been described in a previous work^43^ (where CoA was Co1 and CoC was Co2) and the results of their characterization, together with those of the sample CoB and core shells, are reported in Table 1. A complete list of the sizes and size distribution calculated by different techniques is reported in Table 1S.†

**Table 1:** Cell parameter (*a*), volume-weighted particle size (〈*D*_TEM_V_〉) and standard deviation (SD_*D*_TEM_V_), shell thickness (*Δ*_TEM_) calculated as the difference between 〈*D*_TEM_V_〉 of the core–shell and core, magnetic size (〈*D*_MAG_〉), and M : Fe ratio determined by ICP-AES of the core–shell samples and respective core

Sample	*a* (Å)	〈*D*_TEM_V_〉 (nm)	SD_*D*_TEM_V_ (nm)	Shell thickness *Δ*_TEM_ (nm)	〈*D*_MAG_〉 (nm)	M : Fe^a^
CoA	8.39(1)	5.9	1.0	—	4.4	0.49
CoA@Mn	8.40(2)	9.7	1.1	1.9	4.8	0.41
CoA@Fe	8.36(1)	10.9	1.2	2.5	8.0	—
CoB	8.38(1)	7.5	1.1	—	5.1	0.45
CoB@Mn	8.41(1)	13.2	1.6	2.9	6.3	0.43
CoB@Fe	8.35(1)	12.8	1.7	2.7	6.5	—
CoC	8.38(1)	9.0	1.3	—	5.3	0.55
CoC@Mn1	8.44(9)	13.3	1.7	2.1	6.9	0.41
CoC@Mn2	8.43(1)	14.9	1.6	3.0	7.6	0.45
CoC@Fe1	8.36(9)	11.7	1.5	1.4	8.4	—
CoC@Fe2	8.38(1)	12.8	1.7	1.9	8.2	—

a Referred to the shell fraction in case of core–shell samples.

**Fig. 1:**
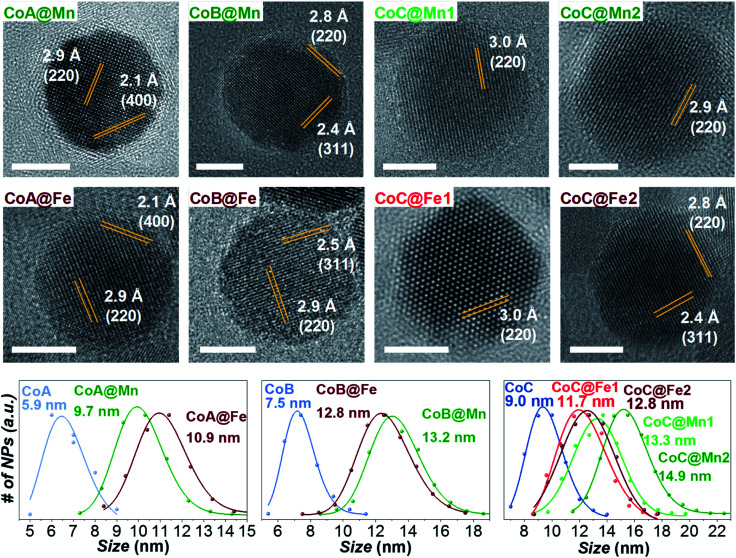
HRTEM images and particle size distributions of the samples.

**Fig. 2:**
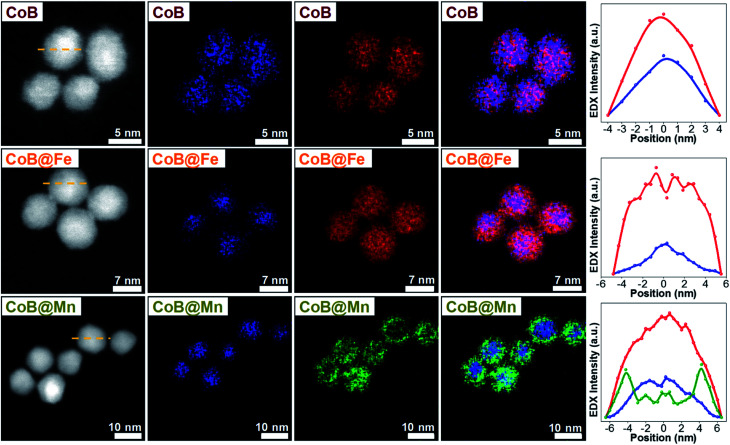
STEM-EDX maps and line profiles across the NPs along the yellow dotted line of the samples CoB, CoB@Fe, and CoB@Mn. Cobalt is represented in blue, manganese in green, iron in red.

As for the CoA and CoC sets of core and core–shell samples,^43^ the other samples (CoB, CoB@Fe, CoB@Mn, CoC@Mn1, CoC@Mn2) showed a XRD pattern typical of a spinel ferrite with no other phases (Fig. 1S and 2S†). The core–shell samples showed larger crystalline size with respect to the core and different cell parameter (*a*), higher for Cox@Mn and lower for Cox@Fe (Table 1). The TEM bright-field images (Fig. 1S and 2S†) revealed spherical NPs with narrow and monomodal size distribution (Table 1 and Fig. 1). HRTEM images (Fig. 1) revealed the high crystallinity of the NPs and epitaxial growth of the shell on the pre-existing cores without lattice mismatches. The formation of the core–shell architecture was proven through nanoscale chemical mapping by STEM-EELS and EDX.^43^Fig. 2 shows the STEM-EDX nanoscale chemical mapping of CoB, CoB@Mn, and CoB@Fe with the corresponding line profile that unambiguously indicated the formation of a core–shell heterostructure through a homogenous coating of the isostructural spinel phase around the core.

**Fig. 3:**
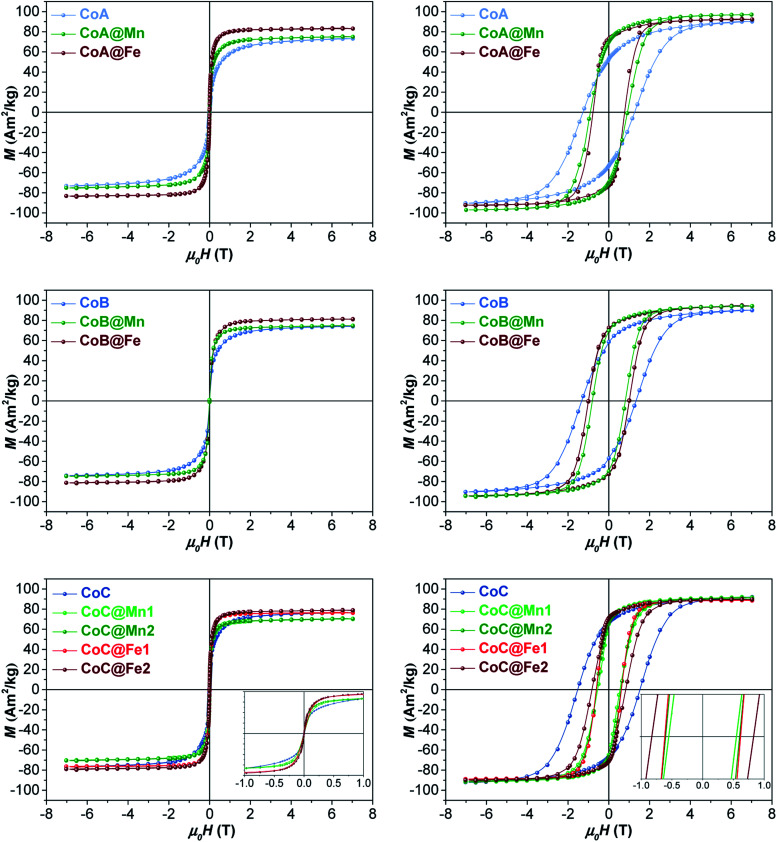
Magnetization isotherms of core–shell samples and respective cores recorded at 300 K (left) and 10 K (right).

**Fig. 4:**
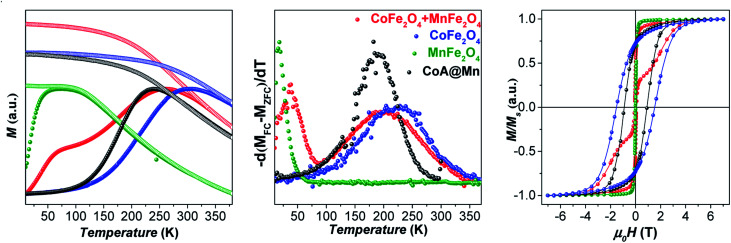
ZFC (full circles) and FC (empty circles) curves, normalized for the magnetization at *T*_max_ of the ZFC curve, recorded at a low external magnetic field (10 mT) of cobalt ferrite of *ca.* 8 nm, manganese ferrite of *ca.* 8 nm, a 1 : 1 w/w mixture of them, and CoA@Mn (left); anisotropy energy barrier distributions estimated by the first derivative −d(*M*_FC_ − *M*_ZFC_)/d*T* (middle); magnetization isotherms recorded at 10 K (right).

As stoichiometry, degree of inversion, and spin canting are crucial in determining the magnetic properties of spinel ferrite NPs, ICP-AES measurements (M : Fe ratios reported in Table 1) and low-temperature (4 K) ^57^Fe Mössbauer spectroscopy in the absence and presence of a magnetic field (6 T) were performed on some selected samples (Fig. 3S, 4S and Table 2S†).

Concerning the cores, almost stoichiometric cobalt ferrite with a degree of inversion of approximately 0.7 was revealed for all samples, in agreement with previous studies.^45,54–58^ A sub-stoichiometry with a Mn : Fe ratio in the range of 0.40–0.45 and a degree of inversion of approximately 0.45 was found for all Cox@Mn samples, in agreement with the trend observed for single manganese ferrite NPs prepared by the same solvothermal method, suggesting the absence of Mn^III^ and indicating a slight preference of Mn^II^ for tetrahedral coordination in all samples.^43,52,53,59^ In the case of Cox@Fe samples, direct information on Fe^II^ : Fe^III^ was not achievable, but through the [Fe]_Oh_/(Fe)_Td_ ratio, it was possible to determine the nature of the iron-based shell (see paragraph entitled “*Low-temperature Mössbauer Spectroscopy*” in ESI for details†). In particular, this ratio should theoretically be 1.67 for maghemite and 2 for magnetite.^60^ The core–shell samples showed values close to 1.67 with higher values for CoB@Fe and CoC@Fe, suggesting that oxidation phenomena took place at the surface (Table 2S†). These findings allowed the shell to be described as primarily composed of maghemite, even though magnetite is also present, especially for the larger core–shell samples (CoB@Fe and CoC@Fe). Moreover, spin canting (Table 2S†) was not revealed in both core and core–shell samples, supporting the evidence from HRTEM data of a homogeneous epitaxial growth of the shell around the core with a formation of a single crystalline domain, as also corroborated by XRD analysis (Table 1S†).

In light of these results, the magnetic properties can be discussed beyond any stoichiometric or structural variability.

**Fig. 5:**
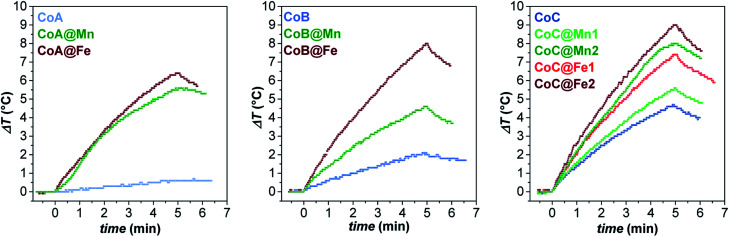
Heating curves of the aqueous colloidal dispersions (*C*_magn_ = 3.4 mg mL^−1^) of all samples at 30 °C, obtained under a magnetic field of 183 kHz and 17 kA m^−1^.

The samples were characterized by DC and AC magnetometry measurements. ZFC-FC protocols and magnetization isotherms at 10 K and 300 K (Fig. 3, 5S and 6S†) were carried out on the cores and core–shell NPs to study the magnetic coupling between the *hard* and *soft* FiM phases. The rigid coupling between the two FiM phases in the core–shell systems was highlighted by (i) the presence of a single-stage hysteresis loop at 10 K (Fig. 3); (ii) a decrease in the coercive and anisotropy fields with respect to the corresponding cores at 10 K (Fig. 3 and Table 2); (iii) the presence of a single dominating maximum in ZFC-FC curves shifted toward higher temperatures, suggesting an increase in effective magnetic anisotropy and/or magnetic diameter (Fig. 5S† and Table 2); (iv) an increase in the magnetic diameter (〈*D*_MAG_〉 estimated by eqn (5S)† at 300 K as reported in the “*DC magnetometry (calculations)*” paragraph in ESI,†Table 1).

**Table 2:** Basic parameters determined from the ZFC-FC curves, magnetization isotherms and temperature dependence of *χ*′ and *χ*″: maximum ZFC temperature (*T*_max_), furcation point of the ZFC-FC curves (2% difference, *T*_diff_), blocking temperature (*T*_b_), coercive field at 10 K (*H*_c_^10^), anisotropy field at 10 K (*H*_K_^10^), saturation magnetization at 10 and 300 K (*M*_s_^10^, *M*_s_^300^), remnant magnetization at 10 K (*M*_r_^10^), magnetic moment (*µ*_m_, median), Néel relaxation time at 300 K (*τ*_N_). *T*_b_ has been calculated as the maximum of the energy barrier distribution (−d(*M*_FC_ − *M*_ZFC_)/d*T*). *H*_k_ has been calculated by considering 2% of the difference between the magnetization and demagnetization curves in the magnetization isotherm at 10 K

Sample	*T*_max_ (K)	*T*_diff_ (K)	*T*_b_ (K)	*H*_c_^10^ (T)	*H*_K_^10^ (T)	*M*_s_^10^ (A m^2^ kg^−1^)	*M*_r_^10^ (A m^2^ kg^−1^)	*M*_r_/*M*_s_	*M*_s_^300^ (A m^2^ kg^−1^)	*µ*_m_ (10^3^*µ*_B_)	*τ*_N_ (s)
CoA	195(3)	270(9)	126(2)	1.28(1)	4.2(1)	90(3)	53(2)	0.55	73(2)	2.6	4 × 10^−7^
CoA@Mn	246(3)	275(3)	185(5)	0.92(1)	2.6(1)	97(4)	67(2)	0.72	75(2)	3.7	4 × 10^−6^
CoA@Fe	294(1)	262(3)	199(3)	0.76(1)	2.0(1)	92(1)	72(2)	0.78	83(3)	12.5	6 × 10^−5^
CoB	241(3)	266(3)	163(2)	1.32(2)	3.8(1)	90(4)	58(3)	0.62	74(3)	3.9	6 × 10^−7^
CoB@Mn	314(3)	312(3)	233(2)	0.81(1)	2.3(1)	94(3)	74(3)	0.73	75(3)	8.3	7 × 10^−4^
CoB@Fe	337(3)	333(3)	237(4)	1.02(1)	2.5(2)	94(3)	72(3)	0.76	81(2)	7.6	5 × 10^−3^
CoC	274(3)	313(3)	206(2)	1.54(1)	4.1(1)	92(1)	67(1)	0.67	77(1)	4.4	4 × 10^−6^
CoC@Mn1	292(2)	295(1)	216(1)	0.56(2)	2.5(1)	92(1)	66(2)	0.69	70(1)	10.8	5 × 10^−5^
CoC@Mn2	348(3)	>380	251(5)	0.60(1)	1.9(1)	91(2)	67(1)	0.72	71(1)	14.5	1 × 10^−3^
CoC@Fe1	278(5)	270(5)	190(1)	0.60(1)	1.8(1)	89(3)	71(2)	0.79	77(3)	15.8	7 × 10^−6^
CoC@Fe2	352(4)	>380	246(4)	0.83(1)	2.6(1)	90(2)	71(1)	0.79	79(2)	14.7	4 × 10^−3^

Furthermore, the comparison between the core–shell systems with a physical reference mixture of cobalt ferrite and manganese ferrite of approximately 8 nm (〈*D*_XRD_〉) in a 1 : 1 mass ratio corroborates this hypothesis. For this sample, as expected, the hysteresis loop and the ZFC-FC curves (Fig. 3 and 4) display the contribution of the two individual spinel ferrite phases. Due to the low thickness of the *soft* shell, the two phases are expected to be rigidly coupled and reverse at the same nucleation field, *H*_n_ (which, as a first approximation is assumed to correspond to the anisotropy field *H*_K_, listed in Table 2),^61^ which depends on the anisotropy constant, saturation magnetization, and volume fraction of the *soft* and *hard* phases.^11^ Once the single-phase magnetic behaviour for all samples and the magnetic coupling in the core–shell samples were ascertained, the DC magnetic measurements together with the temperature dependence of in-phase and out-of-phase susceptibilities (on powders) were investigated to evaluate the critical magnetic parameters known as affecting the heating ability, such as saturation magnetization, magnetic volume, magnetic anisotropy, and Néel relaxation time (see “*Details on magnetic fluid hyperthermia*” paragraph in the ESI, Fig. 7S and 8S†).^62,63^

At 10 K (Fig. 3, right side), the hysteretic behaviour is characterised by saturation magnetization values similar for all samples, in the range 90–100 A m^2^ kg^−1^. The reduced remanent magnetization (*M*_r_/*M*_s_) is around 0.7 for manganese ferrite coated core–shells and 0.8 for spinel iron oxide ones, suggesting a moderate prevalence of cubic to uniaxial anisotropy. Anisotropy field *H*_K_ is around 4 T for cobalt ferrite samples and in the range of 1.8–2.6 T for the core–shell samples, due to the rigid coupling of the *soft*-shell. On the contrary, magnetization isotherms at 300 K (Fig. 3, left side) display a superparamagnetic behaviour for all samples with generally higher saturation magnetization values (*M*_s_^300^, Table 2) for the core–shell NPs with respect to the cores, in agreement with the literature for similar systems.^25,34,35^ The highest saturation magnetization is reached for spinel iron oxide coated core–shell NPs. This is apparently in contrast with the results reported in the literature for similar systems prepared thought a different synthesis strategy,^33^ where higher saturation magnetization values were found for manganese ferrite shells than iron-oxide ones. Such a discrepancy could be related to differences in the formation mechanism of the nanoparticles, strictly dependent on the synthesis method, which may influence stoichiometry of the constituents, oxidation state of the metal(s), degree of inversion, spin canting phenomena, and as a consequence the magnetic properties. Moreover, within each set of samples, the magnetization at a low field (<1 T) and the magnetic energy dissipation are always higher in the Cox@Fe with respect to Cox@Mn, as shown in the insets of Fig. 3. The median magnetic moment (µm, Table 2) of the NPs and the magnetic diameter (〈*D*_MAG_〉, Tables 1 and 3S†) were estimated. 〈*D*_MAG_〉 was found to increase moving from CoA to CoC, in agreement with the increased crystalline and particle sizes. As mentioned before, also an increase of 〈*D*_MAG_〉 was observed in the core–shell samples as expected since a homogenous growth of magnetically coupled phases took place. Moreover, higher values were found for Cox@Fe samples with respect to the Cox@Mn, and for CoC@Mn2 and CoC@Fe2 compared to CoC@Mn1 and CoC@Fe1, respectively, in line with the observed 〈*D*_XRD_〉 and 〈*D*_TEM_〉.

Effective anisotropy constants (*K*) were calculated in different ways, as reported in the “*DC magnetometry*” paragraph of ESI (Table 4S†) and, besides the computation method, in all cases, core–shell systems feature lower anisotropy constant values than those of the respective cores.

Since both anisotropy constants and magnetic volume affect the Néel relaxation time (*τ*_N_), AC magnetometry was used to determine the temperature dependence of the in-phase (*χ*′) and out-of-phase (*χ*″) component of the magnetic susceptibility at different frequencies (0.1–1000 Hz) (Fig. 9S and 10S†) and *τ*_N_ at 300 K estimated by the Vogel–Fulcher equation (Fig. 11S and eqn (17S)†)^64^ is reported in Tables 2 and 5S.†*τ*_N_ are equal to 4 × 10^−7^ s, 6 × 10^−7^ s, and 4 × 10^−6^ s for CoA, CoB, and CoC, respectively, in line with the increased particles' size of the samples. In the same way, *τ*_N_ of core–shell samples is slower than those of the respective cores, due to the increased magnetic volume that dominates the overall decrease of effective anisotropy, as already observed. Generally, spinel iron oxide coated samples feature slower *τ*_N_ with respect to the manganese ferrite coated ones, as well as the thicker shell samples compared to the thinner ones.

Based on the values of *M*_s_^300^, 〈*D*_MAG_〉, *K*, and *τ*_N_, better heating performances should be expected, in order: (i) for the largest core particles, (ii) for the core–shell samples with respect to the corresponding cores, (iii) and for the spinel iron oxide-coated samples with respect to the manganese ferrite ones, (iv) for the samples having the thicker shell than the thinner ones.

In this context, the samples were tested for magnetic heat release in experimental conditions close to those used for clinical applications of magnetic fluid hyperthermia (MFH) in terms of solvent and magnetic field parameters: the measurements were performed on aqueous colloidal dispersions obtained by intercalation with cetyltrimethylammonium bromide (see “*Intercalation process*” paragraph in ESI, Fig. 12S†) keeping the amplitude and frequency of the alternate magnetic field below the clinical threshold.^65^ The heating curves are reported in Fig. 5.

While the CoA sample did not heat up, the larger CoB and CoC NPs provided a sizeable heat release corresponding to SAR values of 21 ± 1 and 32 ± 3 W g_ox_^−1^, respectively (Table 3). In all core–shell systems, a remarkable increase in SAR with respect to the cores alone was observed with values ranging between 20 and 59 W g_ox_^−1^, depending on the chemical nature and thickness of the coating (Table 3).

**Table 3:** Specific absorption rate (SAR) and intrinsic loss power (ILP) values of the core–shell samples and respective core. SAR and ILP are given as the watt per gram of the spinel ferrite phase. * These values indicate a negligible heating release for the sample CoA

Sample	SAR (W g_ox_^−1^)	ILP (nH m^2^ kg_ox_^−1^)
CoA	0*	0*
CoA@Mn	20(1)	0.38(2)
CoA@Fe	42(2)	0.80(4)
CoB	21(1)	0.39(1)
CoB@Mn	27(2)	0.52(4)
CoB@Fe	48(1)	0.92(2)
CoC	32(2)	0.60(4)
CoC@Mn1	43(3)	0.81(6)
CoC@Mn2	47(2)	0.89(4)
CoC@Fe1	46(4)	0.88(8)
CoC@Fe2	59(2)	1.12(4)

The experimental hyperthermic data collected on these three different core–shell series revealed some general aspects:

(1) Core–shell samples heated up more than respective cores;

(2) In all sets, spinel iron oxide-coated core–shell NPs performed better than manganese ferrite-coated ones;

(3) In the CoC series, both systems (CoC@Fe1/CoC@Fe2 and CoC@Mn1/CoC@Mn2) showed that an increase in the shell thickness induces an improvement in the heating abilities.

These results are totally consistent with the previous hypothesis based on the size and magnetic parameters of powdered samples. Nevertheless, to get close to the experimental conditions in which the heating abilities were evaluated, a set of AC magnetic measurements was carried out on the hydrophilic ferrofluids of the CoB series (Fig. 13S†). The temperature dependences of the in-phase and out-of-phase susceptibilities (*χ*′, *χ*″) showed a single maximum (*T*^AC^_max_): below the melting point for the CoB sample and above it for the core–shell ones. A noticeable asymmetry in the curves is visible for all samples indicating different behaviour below and above the melting points, making the estimation of Néel relaxation times more complicated. Indeed, when the solution melts, Brownian motions may occur and also the interparticle interactions may change. Moreover, the *T*^AC^_max_ values of the ferrofluids result to be shifted towards higher temperatures with respect to those of the powdered samples. This behaviour is also observed in the ZFC-FC curves of the CoB dispersion if compared with the CoB powder (Fig. 14S†), in which other features are also visible: a decrease in the broadening of the ZFC peak and an enhanced flatness of the FC curve at temperatures below the *T*_max_. Both the *T*_max_ shift and the enhancement in the flatness of the FC curve are hints of stronger interparticle interactions, which are independent of the ferrofluid's concentration (Fig. 14S†). These findings, even though appear awkward, suggest renormalization of interparticle interactions in the fluid in comparison with the powder and therefore the occurrence of agglomeration/aggregation phenomena which are independent of the concentration. Dipolar interactions most likely occur and cause the formation of secondary entities, whose size (number of primary NPs) and shape (random or controlled clustering such as chain-like alignment) affect the resulting magnetic behaviour and heating ability under an applied static or dynamic magnetic field. In the literature, specific studies on the effect on the heating abilities of the interparticle interactions for spinel ferrite-based core–shell nanoparticles are not available, probably due to the complexity of the systems in which many parameters may affect the magnetic and hyperthermic properties. Concerning single-phase systems, some experimental studies reported the enhancement or reduction of SAR as a function of the dipolar interactions,^66–72^ but other authors^73^ provided a general theoretical model able to explain the heating release behaviour of NPs in the blocked-state, based on their intrinsic magnetic properties (anisotropy, magnetization) and experimental conditions (concentration and magnetic field amplitude). In our case, no changes occur in the strength of dipolar interactions, in the dispersions, in the concentration range 0.7–3.4 mg mL^−1^ (Fig. 14S†). Moreover, also SAR values are independent of interparticle interactions, as reported in Fig. 15S and Table 6S† for a cobalt ferrite sample measured at different concentrations and different capping molecules. Unfortunately, besides this concentration and capping agent independences, it is not possible to speculate on the differences of magnetic properties between powder and colloidal dispersion, the mechanism of formation of secondary entities, and their role in the magnetic properties and in the heating efficiency. Indeed, it has to be taken into account that ferrofluids are dynamic hybrid organic–inorganic systems in which the capping agents might be involved in different processes occurring in liquid phase and feature different physical properties (*e.g.* hydrophobicity for oleate and hydrophilicity for CTAB molecules). For all the above reasons, the evaluation of magnetic parameters from ferrofluids appears to be complicated and not strictly reliable, while the previous discussion of *M*_s_^300^, 〈*D*_MAG_〉, *K*, and *τ*_N_, extracted from measurements on powdered samples, although being a simplification of the system under study, helped in understanding the effect on the heat release performances of the magnetic features of the sole inorganic counterpart, making negligible the influences of the liquid-phase processes.

On the one hand, all the above findings seem to depict a more complex scenario behind the heating abilities of bi-magnetic core–shell systems than simple relationships with single magnetic or microstructural parameter(s). On the other hand, they suggest that a DC/AC magnetic characterization can be helpful in driving the engineering of the heat mediators and they also proved the enhancement of the heating abilities of these heterostructures, especially with a shell of spinel iron oxide, with respect to their corresponding cores in water colloidal dispersions, in principle making these systems biocompatible and promising for application in magnetic heat generation.

## Conclusions

A seed-mediated growth strategy in a solvothermal condition made available three sets of bi-magnetic spinel ferrite core–shell NPs with low size dispersity, allowing the tuning of the core size, chemical nature of the shell, and shell thickness. The effects of these three parameters on the heating abilities were studied for the first time together, revealing unforeseen results on the shell influence. Direct proof of the core–shell structure formation was successfully provided by chemical mapping at the nanoscale using STEM-EDX. The chemical composition, cation distribution, and spin canting were also studied through ICP-AES and low-temperature ^57^Fe Mössbauer spectroscopy, leaving out these features from their influence on the magnetic properties. DC magnetometry demonstrated the rigid coupling between the hard and the soft phases and provided, in combination with the AC magnetometry, the main magnetic parameters theoretically responsible for the heat generation. The availability of several samples allowed the identification of suitable comparisons from which a single parameter-effect emerged.

The heating abilities of the aqueous colloidal dispersions of all samples were tested under mild experimental conditions. For all sets of samples, spinel iron oxide shells featured higher heat release than those of manganese ferrite ones. Moreover, for the first time for hard core-based core–shell NPs, it was observed that the thicker the soft shell, the better the performances. These results were justified in agreement with the hypothesized behaviour based on the magnetic properties, such as saturation magnetization at 300 K, magnetic volume, anisotropy and Néel relaxation time.

The study thus demonstrated the importance of a sophisticated approach based on the synergy of chemical, structural, and magnetic probes down to a single-particle level. Finally, considering the biocompatibility of the iron oxides, this systematic fundamental study proved that a proper design of cobalt ferrite cores coated with a homogenous crystalline shell of spinel iron oxide in principle should lead to biocompatible heat mediators with a net improvement in the heating abilities in comparison with the corresponding cores.

## Conflicts of interest

There are no conflicts to declare.

## Supplementary Material

NA-002-D0NA00134A-s001
