# Supplementary material for: Coupled hard–soft spinel ferrite-based core–shell nanoarchitectures: magnetic properties and heating abilities
Source: Nanoscale Adv. 2020 May 6;2(8):3191–201. doi: 10.1039/d0na00134a (PMC9419663; doi:10.1039/d0na00134a)
Supplement: NA-002-D0NA00134A-s001 [file NA-002-D0NA00134A-s001.pdf]

Electronic Supplementary Material (ESI) for Nanoscale Advances.
This journal is © The Royal Society of Chemistry 2020

Electronic Supplementary Information

for

Coupled Hard-Soft Spinel Ferrite-Based Core-Shell Nanoarchitectures: Magnetic Properties and Heating Abilities

Marco Sanna Angotzi,[‡a,b] *Valentina Mameli,*[‡a,b] *Claudio Cara,*[a,b] *Anna Musinu,*[a,b] *Claudio Sangregorio,*[b,c,d] *Daniel Niznansky,*[e] *Huolin L. Xin,*[f,g] *Jana Vejpravova,*[*e,h] *Carla Cannas*[*a,b,i]

[‡]These authors equally contributed to the work.
[a]Department of Chemical and Geological Sciences, University of Cagliari, S.S. 554 bivio per Sestu, 09042 Monserrato (CA), Italy
[b]Consorzio Interuniversitario Nazionale per la Scienza e Tecnologia dei Materiali (INSTM), Via Giuseppe Giusti 9, 50121 Firenze (FI), Italy
[c]Istituto di Chimica dei Composti OrganoMetallici - Consiglio Nazionale delle Ricerche (ICCOM-CNR), Via Madonna del Piano 10, 50019 Sesto Fiorentino (FI), Italy
[d]Department of Chemistry "U. Schiff", University of Florence, Via della Lastruccia 3-13, 50019, Sesto Fiorentino (FI) Italy
[e]Department of Inorganic Chemistry, Charles University, Hlavova 8, 12800 Prague 2, Czech Republic
[f]Center for Functional Nanomaterials, Brookhaven National Laboratory, 735 Brookhaven Ave, Upton, NY 11973, United States of America
[g]Department of Physics and Astronomy, University of California, Irvine, CA 92697, United States
[h]Department of Condensed Matter Physics, Charles University, Ke Karlovu 5, 12116 Prague 2, Czech Republic
[i]Consorzio per la promozione di Attività Universitarie Sulcis-Iglesiente (AUSI), Centro di Ricerca per l'Energia, l'Ambiente e il TErritorio (CREATE), Palazzo Bellavista Monteponi, 09016 Iglesias (CI), Italy
*Address correspondence to
Jana Vejpravova: jana@mag.mff.cuni.cz
Carla Cannas: ccannas@unica.it

SAMPLES CHARACTERIZATION

Table 1S. Particle diameter determined by different techniques: D_{TEM}, SD_D_{TEM}, and σ_D_{TEM} are the number-weighted median diameter, standard deviation and distribution width obtained from TEM image analysis. D_{TEM_V} is the volume-weighted particle diameter calculated from the number-weighted data, D_{XRD}^{R} is the particle diameter obtained from the Rietveld analysis of the XRD patterns, D_{XRD}^{SPA} is the particle diameter obtained from the single peaks analysis of the XRD patterns with the Scherrer equation as described in reference [1], D_{MAG}^{O} is the particle diameter obtained from the analysis of the magnetization isotherms in generalized Langevin scaling by using Octave, while D_{MAG}^{M} is obtained by MINORIM software.

Sample	D_{TEM} (nm)	SD_D_{TEM} (nm)	σ_D_{TEM}	D_{TEM_V} (nm)	SD_D_{TEM_V} (nm)	D_{XRD}^{R} (nm)	D_{XRD}^{SPA} (nm)	D_{MAG}^{O} (nm)	D_{MAG}^{M} (nm)
**CoA**	5.4	0.9	0.17	5.9	1.0	5.4	5.6	4.4	4.7
**CoA@Mn**	9.4	1.0	0.11	9.7	1.1	7.1	8.1	4.8	6.5
**CoA@Fe**	10.5	1.2	0.11	10.9	1.2	9.0	10.9	8.0	7.7
**CoB**	7.1	1.0	0.14	7.5	1.1	6.3	6.7	5.1	5.1
**CoB@Mn**	12.7	1.5	0.12	13.2	1.6	8.6	9.6	6.3	6.8
**CoB@Fe**	12.2	1.6	0.13	12.8	1.7	8.8	10.1	6.5	6.3
**CoC**	8.5	1.2	0.14	9.0	1.3	7.1	8.0	5.3	5.5
**CoC@Mn1**	12.6	1.6	0.13	13.3	1.7	8.8	9.3	6.9	6.9
**CoC@Mn2**	14.4	1.6	0.11	14.9	1.6	9.1	11.1	7.6	8.4
**CoC@Fe1**	11.1	1.5	0.13	11.7	1.5	10.2	10.8	8.4	8.3
**CoC@Fe2**	12.1	1.6	0.13	12.8	1.7	10.0	12.1	8.2	9.0

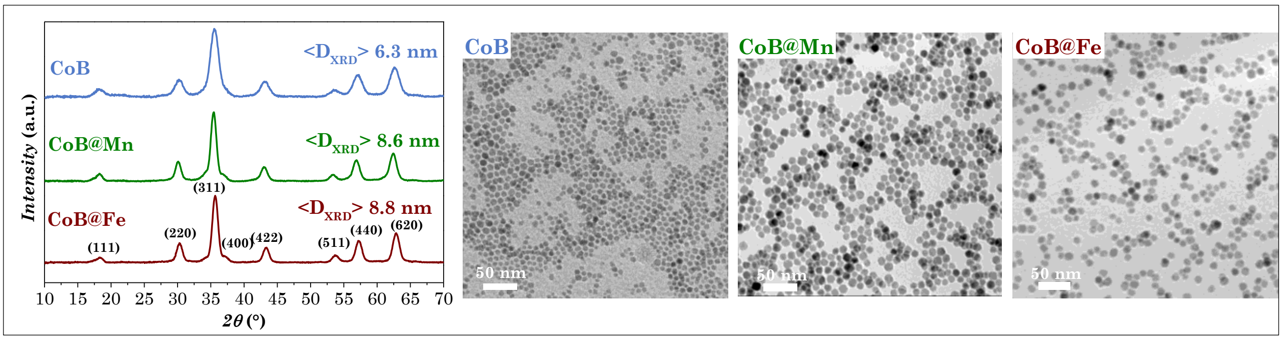

Figure 1S. XRD patterns (left) and TEM (right) of samples CoB, CoB@Mn and CoB@Fe.

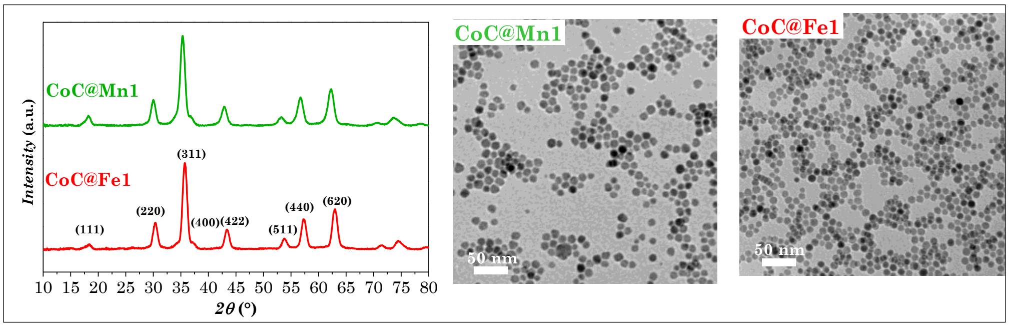

Figure 2S. XRD patterns (left) and TEM (right) of samples CoC@Mn1 and CoC@Fe1.

LOW-TEMPERATURE MÖSSBAUER SPECTROSCOPY.

The in-field measurements were done in a perpendicular arrangement of the external magnetic field with respect to the γ-beam and are useful to get information about the cationic distribution and the canting phenomena in the spinel structure.

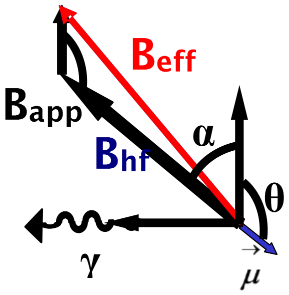

Indeed, the angle θ between the magnetic moment ($\vec{\mu}$) and the applied magnetic field has been estimated thanks to the following equation:

$$B_{eff}^2 = B_{hf}^2 + B_{app}^2 - 2B_{hf}B_{app}\cos\theta \qquad \textbf{Eq. 1S}$$

where B_{hf} is the hyperfine field (B_{hf}^{0T}), B_{eff} the total effective magnetic field at the nucleus (B_{hf}^{6T}), B_{app} the external applied magnetic field, and α is the angle between B_{eff} and B_{app}.

The angle θ corresponds to the canting angle of the magnetic moment for the octahedral sites, whereas for the tetrahedral ones the canting angle is equal to π-θ. This is because of the relative arrangement of the hyperfine and applied fields vectors that are parallel or antiparallel aligned for tetrahedral or octahedral sites, respectively.[2]

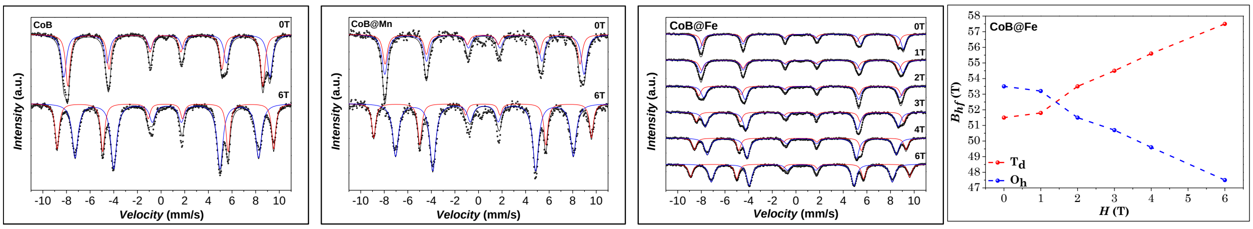

Figure 3S. Low temperature ^{57}Fe Mössbauer spectra with no external magnetic field (upper side) and in the presence of external magnetic field (lower side) of the samples CoB (left), CoB@Mn (middle), and CoB@Fe (right). Octahedral sites are represented in blue, tetrahedral in red. For CoB@Fe, the external field increases up to 6 T.

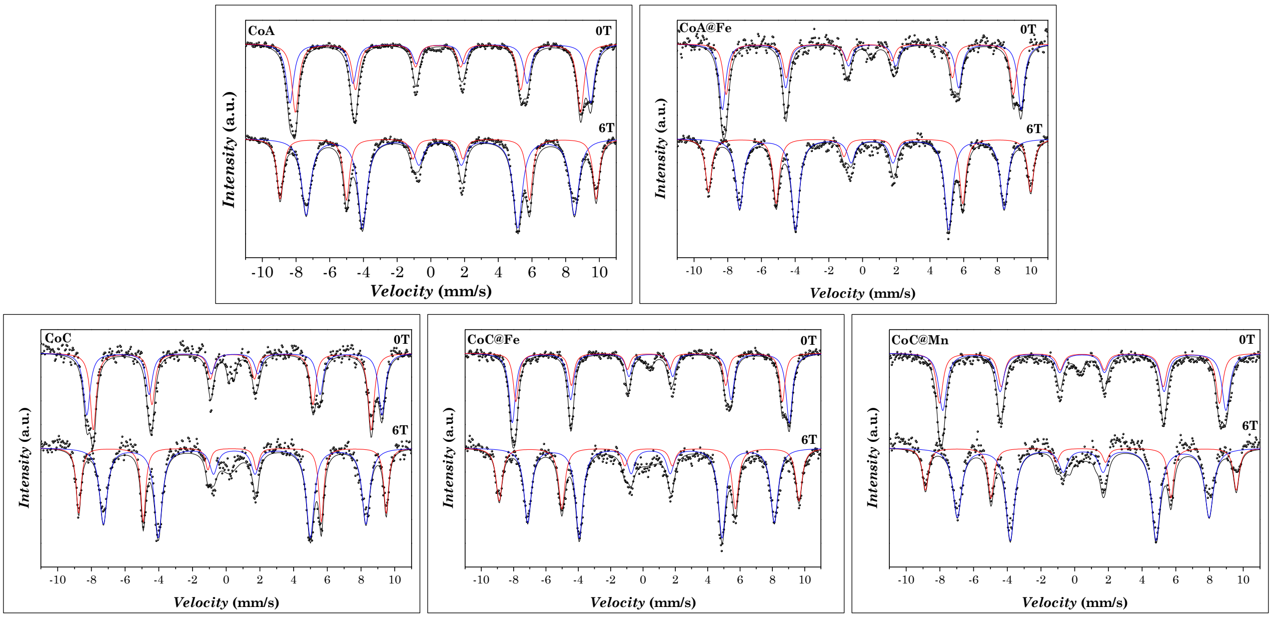

Figure 4S. Low temperature ^{57}Fe Mössbauer spectra with no external magnetic field (upper side) and in the presence of external magnetic field (lower side) of the CoA, CoA@Fe, CoC, CoC@Mn, CoC@Fe samples.

Table 2S. ^{57}Fe Mössbauer parameters of the samples obtained from the spectra recorded at low temperature (4 K) and with a 6 T magnetic field applied: values of the isomer shift (δ), effective field at 0 T ($B_{eff}^{0T} = B_{hf}$) and 6 T (B_{eff}^{6T}), relative area (A), canting angles (α) and chemical formula calculated from site occupancy corrected by ICP-OES data.

Sample	Signal	Site	δ (mm/s)	B_{eff}^{0T} (T)	B_{eff}^{6T} (T)	A (%)	α (°)	γ	Formula
CoA	Sextet	T_d	0.43(1)	52.6(1)	58.3(1)	34%	19	0.67	$(Co_{0.31}Fe_{0.69})[Co_{0.67}Fe_{1.32}]O_4$
	Sextet	O_h	0.56(1)	55.4(1)	49.4(1)	66%	0		
CoA@Fe	Sextet	T_d	0.41(1)	53.0(1)	59.4(1)	37%	0	-	$@(Fe_1)[Fe_{1.65}]O_4$
	Sextet	O_h	0.56(1)	55.1(1)	48.8(1)	63%	0		
CoB	Sextet	T_d	0.37(1)	51.2(1)	56.9(1)	35%	19	0.65	$(Co_{0.27}Fe_{0.72})[Co_{0.65}Fe_{1.33}]O_4$
	Sextet	O_h	0.49(1)	54.4(1)	48.2(1)	65%	10		
CoB@Fe	Sextet	T_d	0.36(1)	51.5(1)	57.5(1)	36%	0	-	$@(Fe_1)[Fe_{1.71}]O_4$
	Sextet	O_h	0.49(1)	53.5(1)	47.5(1)	64%	0		
CoB@Mn	Sextet	T_d	0.36(1)	51.1(1)	57.4(1)	30%	0	0.44	$@(Mn_{0.48}Fe_{0.50})[Mn_{0.44}Fe_{1.55}]O_4$
	Sextet	O_h	0.50(1)	52.5(1)	46.9(1)	70%	20		
CoC	Sextet	T_d	0.37(1)	51.2(1)	56.7(1)	35%	25	0.74	$(Co_{0.33}Fe_{0.68})[Co_{0.74}Fe_{1.27}]O_4$
	Sextet	O_h	0.49(1)	54.4(1)	48.4(1)	65%	0		
CoC@Fe2	Sextet	T_d	0.36(1)	51.2(1)	57.6(1)	36%	0	-	$@(Fe_1)[Fe_{1.74}]O_4$
	Sextet	O_h	0.48(1)	53.2(1)	47.4(1)	64%	14		
CoC@Mn2	Sextet	T_d	0.37(1)	51.2(1)	57.3(1)	27%	0	0.46	$@(Mn_{0.43}Fe_{0.55})[Mn_{0.46}Fe_{1.52}]O_4$
	Sextet	O_h	0.51(1)	52.6(1)	46.4(1)	73%	0		

Without the external magnetic field, the spectra showed the overlapping of two sextets associated with the octahedral and tetrahedral sites of the spinel structure. The in-field measurements allowed us to split these two subspectra and to calculate the occupancy in the two sublattices. For instance, from the relative areas of the

two sextets in the in-field spectrum of CoA, it was found that 34% of iron cations are located in the tetrahedral position and 66% in octahedral positions. Taking into account these results, the inversion degree (percentage of divalent cations in octahedral position) is 0.67 and the formula can be written as $(Co_{0.31}Fe_{0.69})[Co_{0.67}Fe_{1.32}]O_4$, in agreement with the expected random Co^{II} site occupancy in cobalt ferrite nanoparticles.[3–9] Similar results were found for the sample CoA and CoC, whose inversion degrees are equal to 0.65 and 0.74, respectively. For the sample CoA@Fe, it was found from relative areas that 37% of Fe cations are located in tetrahedral positions and 63% in octahedral positions. The comparison of these data with the core ones allowed to estimate the cation distribution of the shell. Taking into account the iron fraction in the core calculated from ICP-OES measurements (0.10) and the site occupancy of the core, we estimated the amount of Fe in the shell, which corresponds to 38% and 62% for tetrahedral and octahedral sites respectively. Consequently, the ratio $Fe(O_h)/Fe(T_d)$ is 1.65. This result is in agreement with the theoretical maghemite ratio, which value is 1.67, while for stoichiometric magnetite is 2.[10] The same behaviour is revealed in the samples CoB@Fe and CoC@Fe2, whose $Fe(O_h)/Fe(T_d)$ ratios are equal to 1.71 and 1.74, respectively. The increased $Fe(O_h)/Fe(T_d)$ ratio with the NPs size is in line with the RT Mössbauer data that suggested a lower degree of oxidation for the larger sample, CoC@Fe2.[1]

By using the same procedure, Fe content in CoB@Mn shell was estimated, and the inversion degree was found equal to 0.46. Consequently, the formula of the shell can be written as $(Mn_{0.43}Fe_{0.55})[Mn_{0.46}Fe_{1.52}]O_4$. Similar behaviour was observed for sample CoC@Mn, with an inversion degree of 0.44. This result is in good agreement with the theoretical value of inversion degree for nanosized manganese ferrite.[11] The chemical formula of the different samples with site occupancies are reported in **Table 2S**.

By using **Eq. 1S**, it is also possible to calculate the canting angles. For sample CoA, the values for the tetrahedral and octahedral sites are 19° and 0°, respectively. Within the experimental error, we can consider that the magnetic moments of both sublattices are not canted being the angles calculated from the cosine's equation (**Eq. 1S**), and therefore small changes in the cosθ lead to significant changes on the angle values. The same results were found for samples CoB and CoC, which canting angle values for tetrahedral and octahedral sites are equal to 19°-10° and 25°-0°, respectively (**Table 2S**). Spin canting has not also been revealed in the core-shell samples.

The sample CoB@Fe was measured at different magnetic fields (from 1 to 6 T) to gather information on the spin saturation process (**Figure 3S**, right). Even with the external magnetic field of 1 T, the splitting of the two sextets was observed. At 0 T the octahedral sites have a larger hyperfine field than tetrahedral ones, while at 6 T an inversion occurred (**Figure 3S**). This is due to the antiparallel direction of the octahedral hyperfine field with respect to the externally applied field. The increase of the hyperfine field in the tetrahedral sites (or decrease in octahedral sites), when a magnetic field of 1 T is applied, is equal to 0.3 T. When a 2 T field is applied, the B_{hf} changes by 1.5 T for T_d and 1.7 T for O_h sites, while it changes of about 1 T for all the subsequent increase of external magnetic field up to 6 T. The different behaviour observed below and above 2 T is probably caused by the unsaturated magnetic moment, that requires a field of such strength to be saturated, as it can be revealed from the field dependence of the magnetization at low temperature (**Figure 3**).

DC MAGNETOMETRY

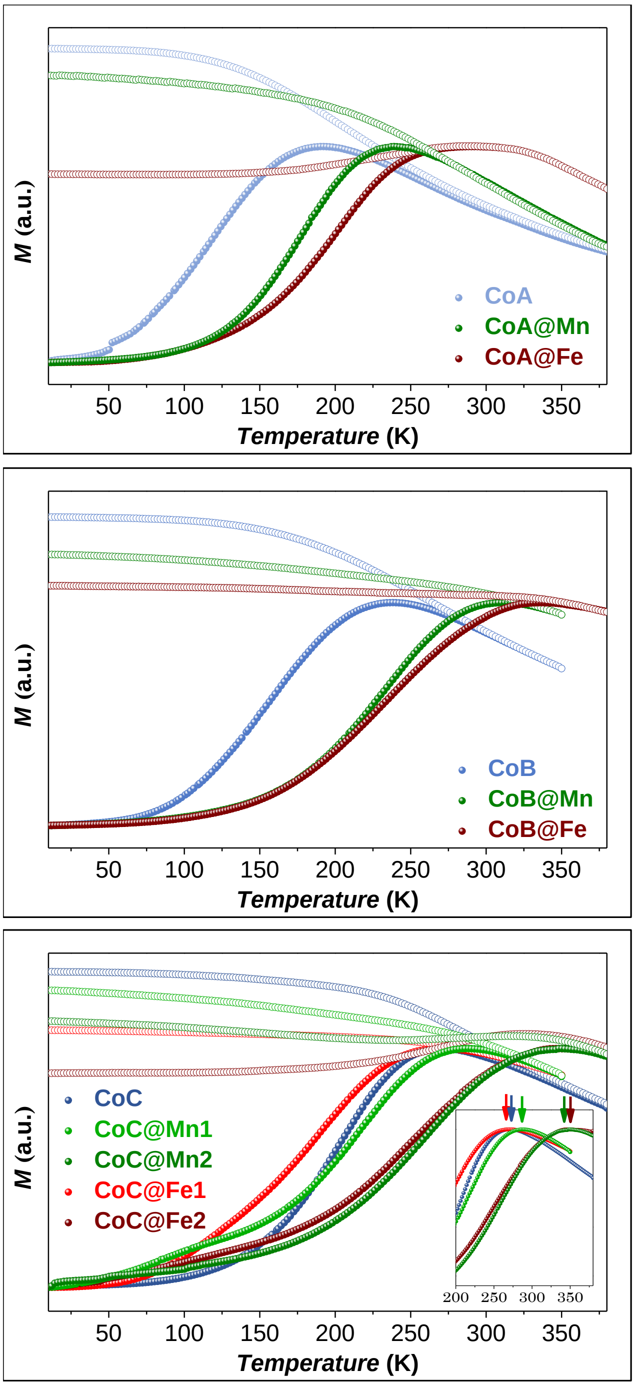

Figure 5S. ZFC (full circles) and FC (empty circles) curves, normalized for the magnetization at T_{max} of the ZFC curve, of core-shell samples and respective cores recorded at low external magnetic field (10 mT).

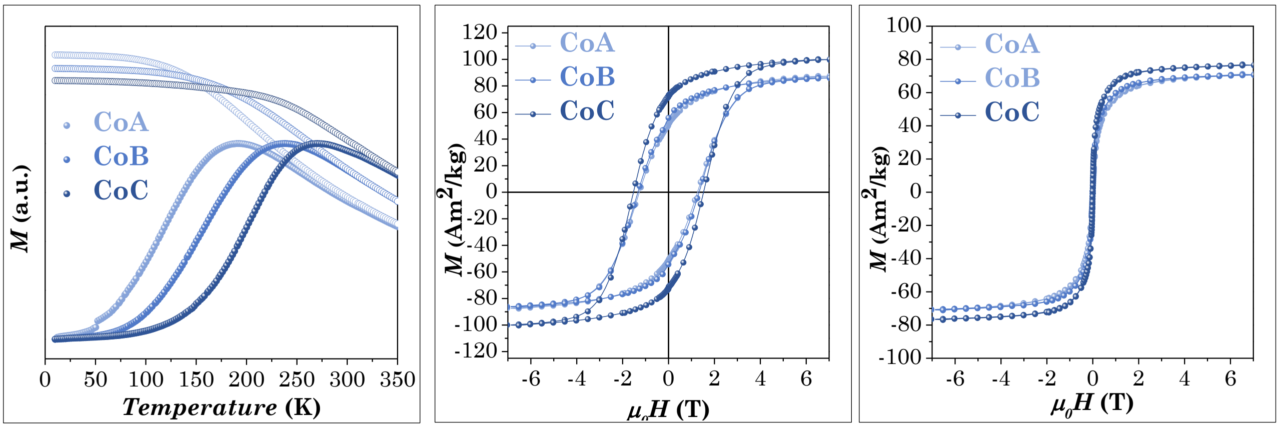

Figure 6S. ZFC (full circles) and FC (empty circles) curves, normalized for the magnetization at T_{max} of the ZFC curve recorded at low external magnetic field (10 mT) (left); Magnetization isotherms recorded at 10 K (middle) and 300 K (right) of cobalt ferrite samples.

ZFC-FC curves of cobalt ferrite cores (**Figure 5S**, **Figure 6S**) show a furcation at a specific temperature (T_{diff}), with a maximum on the ZFC curve (T_{max}), that is proportional to the blocking temperature of the largest particles and the mean value, respectively. T_b is the blocking temperature calculated with the first derivative of the difference curve (M_{FC}-M_{ZFC}) as the temperature at which 50% of the nanoparticles are in the superparamagnetic state.[12,13] Both T_{max} and T_b increases with the size of cobalt ferrite, as predicted by the Stoner-Wohlfarth model,[14] and are in a good agreement with the previously reported values for cobalt ferrite of similar size.[15–18] The saturation of the low-temperature part of the FC curve extends to higher temperatures for larger particles; it is a typical signature of enhancement of inter-particle interactions due to the increase of the mean magnetic moments per particle and reform of effective magnetic anisotropy.[19]

ZFC curves (**Figure 5S**) of core-shell samples show a dominant maximum (T_{max}), associated with majority particle population, also confirmed by a single energy barrier distribution (-d(M_{FC}-M_{ZFC})/dT)[20] centred at a specific temperature (T_b). Both T_{max} and T_b values increase in the core-shell samples compared to the cores, due to the increased particle magnetic volume. The difference T_{diff} - T_{max} is generally lower in the core-shell samples than in the cores, suggesting a decrease in the energy barrier distribution dispersity, because of the homogeneous growth on the shells around the seeds that induces a narrower size distribution.

Nevertheless, also the increase of interparticle interactions in the core-shell systems can affect T_{diff}, as also evidenced by the flatness of the FC curves. In particular, spinel iron oxide coated core-shells show a more pronounced FC saturation, typical for strongly interacting systems (e.g. superspin glass).[21–23]

Magnetization isotherms of cobalt ferrite samples show no hysteretic behaviour at 300 K (**Figure 6S**), typical for particles in the superparamagnetic state. A large hysteresis is instead present at 10 K for cobalt ferrite samples. Coercive field increases with increases the particle size, in agreement with the Stoner-Wohlfarth model,[14] while the saturation magnetization is constant within the experimental error. The reduced remanent magnetization (M_r/M_s) increases with the size of cobalt ferrite, going from 0.55 for CoA to 0.67 for CoC. The values are far from those expected for pure cubic anisotropy (0.83 for bulk cobalt ferrite) and suggest that the cobalt ferrite samples have mixed cubic-uniaxial anisotropy with the first component becoming more dominant with the increase of the particles' size.

For the real system of the superparamagnetic NPs with a size distribution, the magnetization, M of the NPs in the magnetic field, H can be written as a weighted sum of the Langevin functions:

$$M(H,T) = \int_0^\infty \mu L\left(\frac{\mu H}{k_B T}\right) f(\mu) d\mu + \chi_{linear} H \quad \textbf{Eq. 2S}$$

where $f(\mu)$ corresponds to the unimodal log-normal distribution of the magnetic moments, μ expressed as:

$$f(\mu) = \frac{1}{\sqrt{2\pi}\mu\sigma} \exp\left(-\frac{\ln^2\left(\frac{\mu}{\mu_0}\right)}{2\sigma^2}\right) \quad \textbf{Eq. 3S}$$

$$\mu_m = \mu_0 \exp\frac{\sigma^2}{2} \quad \textbf{Eq. 4S}$$

where σ is the distribution width, μ_0 and μ_m are the median and mean magnetic moment, respectively (**Table 3S**). The second term in the equation (**Eq. 2S**) corresponds to an additional linear contribution to the magnetization, which can originate from some diamagnetic or paramagnetic components of the sample. The parameters of $f(\mu)$ were obtained from the refinement of the magnetization isotherm measured above T_B in the Matlab/Octave software.
The median magnetic size, d_{mag} of the particle was calculated from the μ_0 using the expression:

$$D_{MAG} = \sqrt[3]{\frac{6\mu_0 a^3}{\mu_{uc}\pi}} \quad \textbf{Eq. 5S}$$

where a and μ_{uc} are the lattice parameter and the magnetic moment of the unit cell of the spinel phase (calculated assuming site occupancy estimated from LT ^{57}Fe Mössbauer spectroscopy), respectively (**Table 3S**). For comparison, magnetic moments (μ_m) and magnetic diameters (D_{MAG}) have been calculated also by MINORIM software, which uses a non-regularized method.[24] The parameters used for the calculation of magnetic diameters are reported in **Table 3S**.

Table 3S. Cell parameter (a), magnetic moment of the unit cell (μ_{UC}), median magnetic moment, and magnetic diameters calculated by OCTAVE (μ_m^O, D_{MAG}^O) and MINORIM (μ_m^M, D_{MAG}^M).

Sample	a (Å)	μ_{UC} (10^3 μ_B)	μ_m^O (10^3 μ_B)	D_{MAG}^O (nm)	μ_m^M (10^3 μ_B)	D_{MAG}^M (nm)
CoA	8.39	33.8	2.6	4.4	3.1	4.7
CoA@Mn	8.40	38.8	3.7	4.8	9.3	6.5
CoA@Fe	8.36	27.1	12.5	8.0	10.9	7.7
CoB	8.38	33.5	3.9	5.1	3.9	5.1
CoB@Mn	8.41	39.2	8.3	6.2	10.9	6.8
CoB@Fe	8.35	30.4	7.6	6.5	6.8	6.3
CoC	8.38	33.4	4.4	5.3	5.0	5.5
CoC@Mn1	8.44	38.0	10.8	6.9	10.8	6.9
CoC@Mn2	8.43	37.7	14.5	7.6	19.7	8.4
CoC@Fe1	8.36	30.4	15.8	8.3	15.8	8.3
CoC@Fe2	8.38	30.1	14.7	8.2	19.7	9.0

In the literature, effective anisotropy constants are calculated in different ways, summarized below:

$$K_1 = \frac{H_c M_s}{0.96\left(1-\left(\frac{T}{T_b}\right)^{0.77}\right)}$$ **Eq. 6S**[25]

$$K_2 = a(H_K M_s)$$ **Eq. 7S**[26]

where a is equal to 0.5 and 0.64 for uniaxial (K_{2_uni}) and cubic (K_{2_cub}) anisotropy, respectively.

$$K_3 = \frac{25 K_B T_b}{V}$$ **Eq. 8S**[5]

Eq. 6S and **Eq. 7S** depend on saturation magnetization and coercive field or anisotropy field, respectively, and give an approximated value of anisotropy constant assuming collinear orientation with respect to the magnetic field. **Eq. 8S** derives from the energy barrier equation, and it depends on blocking temperature (T_b) estimated from $-d(M_{FC}-M_{ZFC})/dT$ curves, and particle volume anisotropy constant K_3 was calculated by using $<D_{MAG}>$, $<D_{XRD}>$, and $<D_{TEM}>$ values. The results are reported in **Table 4S**.

Table 4S. Anisotropy constant calculated by using Eq. 6S, 7S, and 8S.

**Sample**	K_1 **(10^4 J/m^3)**	K_{2_uni} **(10^4 J/m^3)**	K_{2_cub} **(10^4 J/m^3)**	$K_{3\_}D_{MAG}$ **(10^4 J/m^3)**	$K_{3\_}D_{XRD}$ **(10^4 J/m^3)**	$K_{3\_}D_{TEM}$ **(10^4 J/m^3)**
CoA	75	100	130	10.8	5.9	4.5
CoA@Mn	52	63	81	1.2	3.8	1.5
CoA@Fe	41	40	51	2.8	2.8	1.1
CoB	75	91	120	9.0	4.8	2.8
CoB@Mn	44	55	71	6.8	2.7	0.7
CoB@Fe	56	60	77	6.3	2.5	0.8
CoC	89	100	130	10.1	4.2	2.1
CoC@Mn1	30	59	75	4.8	2.3	0.7
CoC@Mn2	32	44	57	4.2	2.4	0.6
CoC@Fe1	31	40	51	2.3	1.3	0.9
CoC@Fe2	43	59	75	3.3	1.8	0.9

K_1 and K_2 values are in the order of 10^5 J·m^{-3} and are comparable, while K_3 values are one or two orders of magnitude lower, due to the different model adopted for the estimation. Nevertheless, the trend in the core-shell systems is comparable in all cases. Indeed, core-shell samples have generally lower anisotropy constant values compared with the respective cores.

DETAILS ON MAGNETIC FLUID HYPERTHERMIA

Theoretical Calculation of SAR

The equations for the calculation of SAR are as follows:

$$SAR = \frac{P}{\rho\varphi}$$

P is the lost power, expressed as follows:

$$P = \mu_0 \pi \chi'' f H_0^2 \quad \textbf{Eq. 9S}$$

χ'' is the out-of-phase component of the susceptibility, expressed as follows:

$$\chi'' = \frac{\omega\tau}{1 + (\omega\tau)^2}\chi_0 \quad \textbf{Eq. 10S}$$

χ_0 is the actual susceptibility, while τ the effective relaxation time, expressed in **Eq. 11S** and **14S**, respectively.

$$\chi_0 = \chi_i \frac{3}{\xi}\left(coth\xi - \frac{1}{\xi}\right) \quad \textbf{Eq. 11S}$$

χ_i is the susceptibility calculation parameter, and ξ is the Langevin parameter, expressed in **Eq. 12S** and **13S**, respectively.

$$\chi_i = \frac{\mu_0 \varphi M_d^2 V_m}{3 k_B T} \quad \textbf{Eq. 12S}$$

$$\xi = \frac{\mu_0 M_d H_0 V_m}{k_B T} \quad \textbf{Eq. 13S}$$

$$\tau = \frac{\tau_N \tau_B}{\tau_N + \tau_B} \quad \textbf{Eq. 14S}$$

τ_N and τ_B are the Nèel and Brown relaxation times, expressed in **Eq. 15S** and **16S**, respectively.

$$\tau_N = \tau_0 e^{\frac{KV_M}{k_B T}} \quad \textbf{Eq. 15S}$$

$$\tau_B = \frac{3\eta V_H}{k_B T} \quad \textbf{Eq. 16S}$$

The variables are:

- μ_0 = permeability in free space: 1.26 x 10^{-6} (T m A^{-1})
- f = frequency of magnetic field: 183000 (s^{-1})
- H_0 = magnetic field intensity: 17000 (A m^{-1})
- ω = angular frequency: 1.15 x 10^{6} (s^{-1})
- φ = nanoparticle volume fraction: 6.42 x 10^{-4}
- ρ = nanoparticle density: 5.30 x 10^{6} (g m^{-3})
- M_d = magnetization domain: 4.24 x10^{5} (A m^{-1})
- V_m = nanoparticle volume (m^3)
- k_B = Boltzmann constant: 1.38064852 x 10^{-23} (J K^{-1})
- T = temperature: 303.15 (K)
- K = magnetic anisotropy constant (J/m^3)
- η = viscosity of solution: 8.94 x 10^{4} (Pa s)
- V_H = nanoparticles' hydrodynamic volume (m^3): 6 nm exceeding the nanoparticles' diameter.

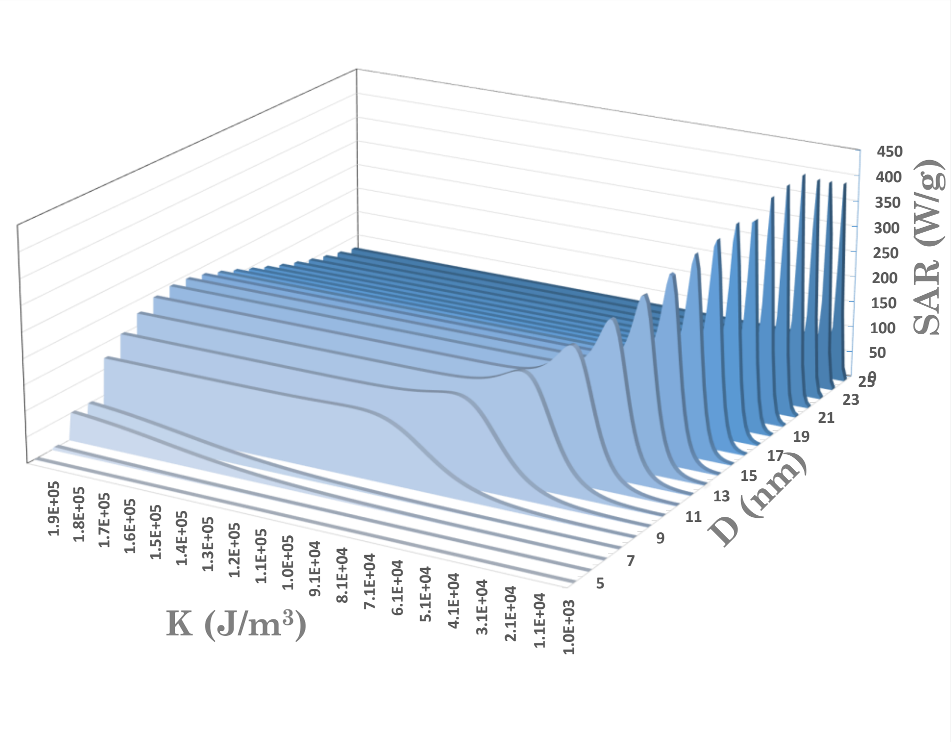

Figure 7S. Simulated plot of SAR based on nanoparticle size (D between 5-25 nm) and magnetic anisotropy constant (K between $1x10^3$ and $2x10^5$ J/m^3) at a magnetization value M_s of 80 emu/g**.**

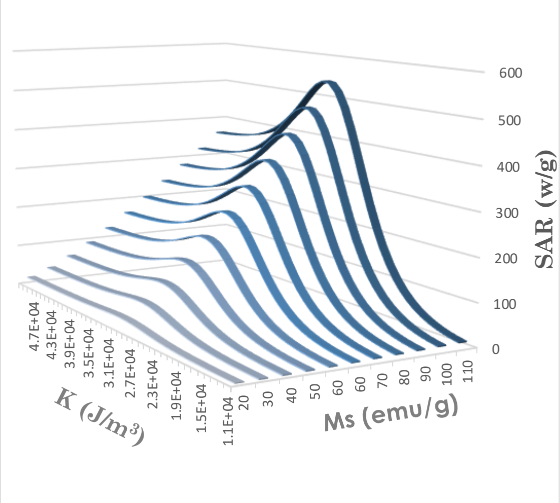

Figure 8S. Simulated plot of SAR based on magnetic anisotropy constant (K between $1x10^3$ and $2x10^5$ J/m^3) and magnetization value (M_s between 20 and 120 emu/g) for 13 nm nanoparticle.

AC MAGNETOMETRY ON POWDERED SAMPLES

AC magnetometry was used to measure the temperature dependence of the in-phase (χ') and out-of-phase (χ'') component of the magnetic susceptibility at different frequencies (0.1-1000 Hz) for the core (**Figure 9S**) and core-shell samples (**Figure 10S**) and the Néel relaxation time estimated by the Vogel-Fulcher equation (**Eq. 17S**,) is reported in **Table 5S**. The Néel relaxation times of core-shell samples are slower than those of the respective cores, due to the increased particle volume that dominates the overall decrease of effective anisotropy.

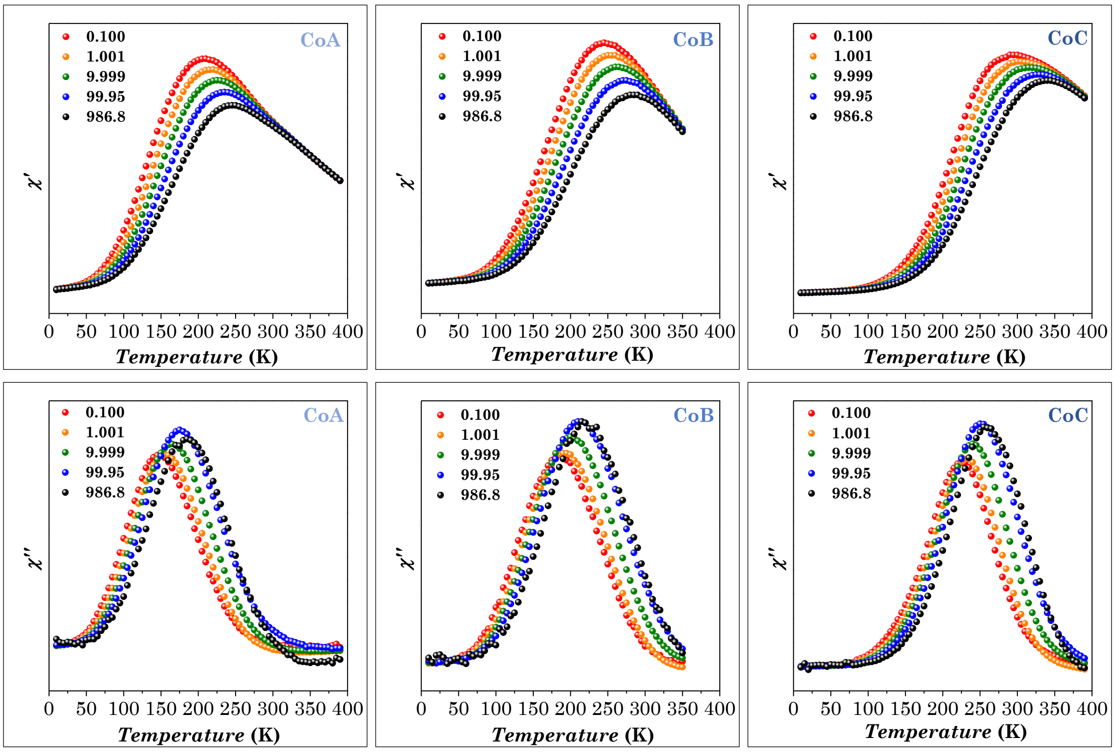

Figure 9S. AC susceptibility measurements of the s CoA, CoB, and CoC samples. The in-phase (*χ'*) component of the magnetic susceptibility is displayed in the upper part and the out-of-phase (*χ''*) in the bottom part.

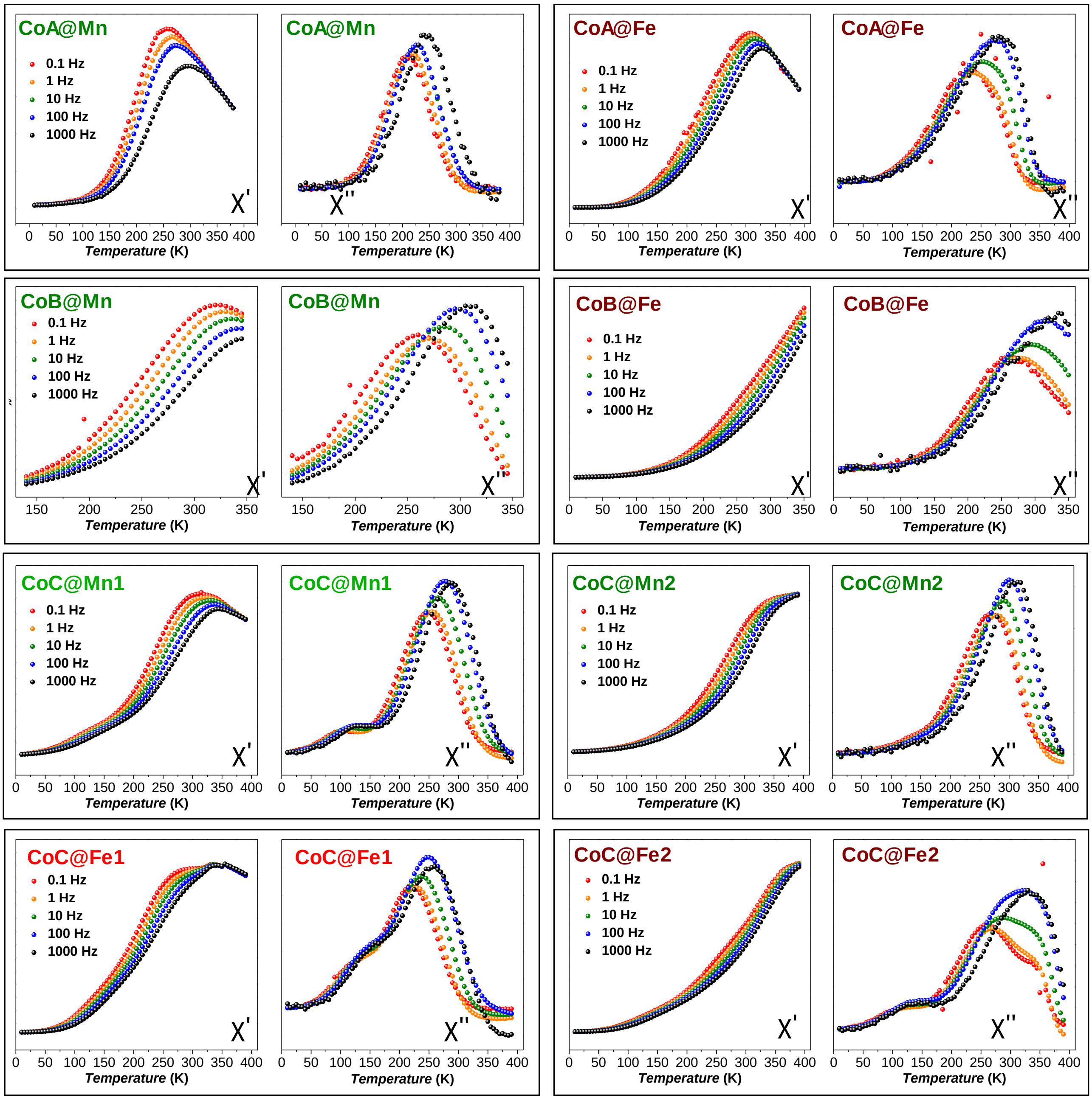

Figure 10S. AC susceptibility measurements of the core-shell samples.

Table 5S. Parameters obtained from the curve fitting by the Vogel-Fulcher model for T = 300 K. τ_0 is the characteristic relaxation time, E_b is the energy barrier against the magnetization reversal, T_0 is the temperature value accounting for the strength of magnetic interactions, τ_N is the Néel relaxation times.

Sample	τ_0 (s)	E_b (K)	T_0 (K)	τ_N (s)
CoA	$3.9 \cdot 10^{-9}$	949	101	$4 \cdot 10^{-7}$
CoA@Mn	$1.1 \cdot 10^{-9}$	1136	163	$4 \cdot 10^{-6}$
CoA@Fe	$1.4 \cdot 10^{-9}$	1559	155	$6 \cdot 10^{-5}$
CoB	$1.0 \cdot 10^{-9}$	1050	136	$6 \cdot 10^{-7}$
CoB@Mn	$8.2 \cdot 10^{-10}$	1515	190	$7 \cdot 10^{-4}$
CoB@Fe	$2.4 \cdot 10^{-9}$	1838	174	$5 \cdot 10^{-3}$
CoC	$7.5 \cdot 10^{-10}$	1092	174	$4 \cdot 10^{-6}$
CoC@Mn1	$6.5 \cdot 10^{-10}$	1224	191	$5 \cdot 10^{-5}$
CoC@Mn2	$5.5 \cdot 10^{-10}$	1325	209	$1 \cdot 10^{-3}$
CoC@Fe1	$7.8 \cdot 10^{-9}$	846	176	$7 \cdot 10^{-6}$
CoC@Fe2	$6.7 \cdot 10^{-9}$	1755	169	$4 \cdot 10^{-3}$

Vogel-Fulcher equation:[27]

$$\tau_N = \tau_0 e^{\left(\frac{E_b}{k_b(T-T_0)}\right)}$$ **Eq. 17S**

Where τ_0 is the characteristic relaxation time, E_b the energy barrier against magnetization reversal, T the absolute temperature, and T_0 the temperature value accounting for the strength of magnetic interactions.

As an example, the calculation for the CoA sample is reported. The Vogel-Fulcher equation (**Eq. 17S**) has been written in the logarithmic form (**Eq. 18S**):

$$\frac{1}{(\ln f - \ln f_0)} = \frac{T}{A} - \frac{T_0}{A}$$ **Eq. 18S**

Where f is equal to $1/2\pi\tau$, f_0 is equal to $1/2\pi\tau_0$, and A correspond to E_b/K_b. As a first approximation, τ_0 has been assumed equal to $1 \cdot 10^{-10}$ (**Figure 11SA**) and $1 \cdot 10^{-8}$ (**Figure 11SB**), to estimate values for T_0, which correspond to 92 K and 110 K, respectively. Then, the averaged T_0 (101 K) has been used to extrapolate the value of A (949 J/K) and τ_0 ($3.7 \cdot 10^{-9}$ s) (**Figure 11SC**), following **Eq. 19S**:

$$\ln f = \ln f_0 + A \frac{1}{(T_{max} - T_0)}$$ **Eq. 19S**

Subsequently, the value A (949 J/K) from **Eq. 19S** has been used to calculate T_0 (101 K) and τ_0 ($4.0 \cdot 10^{-9}$ s) (**Figure 11SD**), following **Eq. 19S**. The τ_0 value calculated from the fitting reported in **Figure 11SC** and **Figure 11SD** (always very close) has been averaged ($3.9 \cdot 10^{-9}$ s).

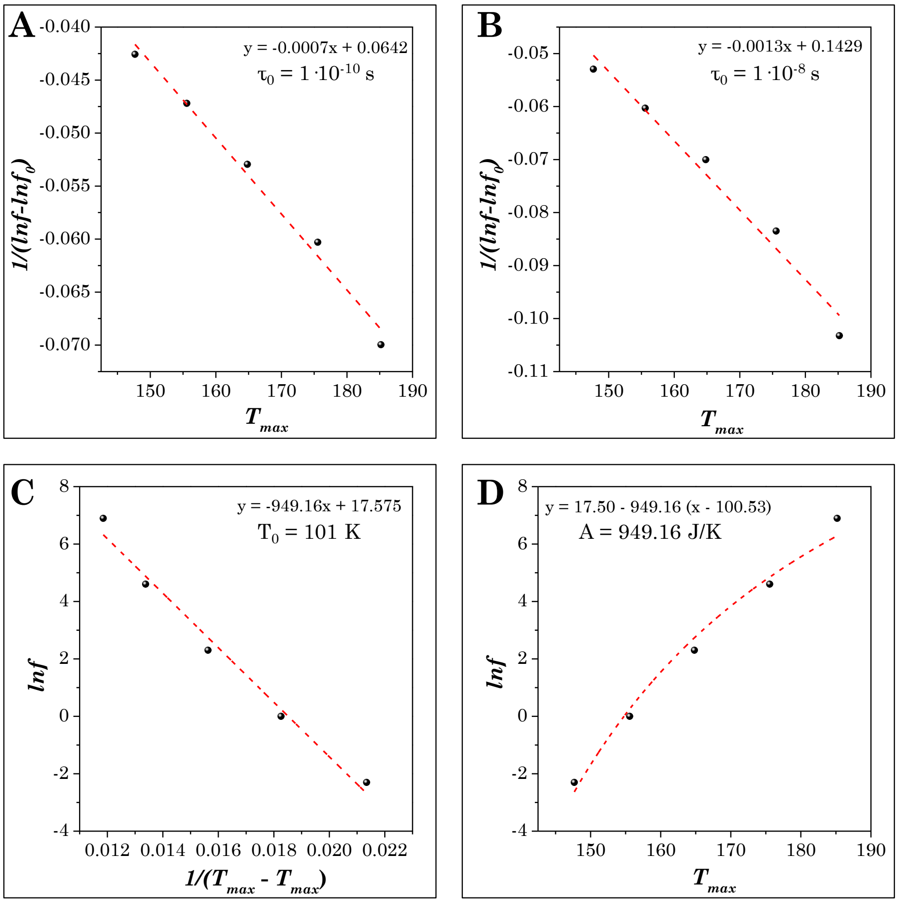

Figure 11S. Curve fitting for the estimation of τ_0, T_0, and KV/K_b by the Vogel-Fulcher model for the CoA sample.

INTERCALATION PROCESS

The hydrophobic nanoparticles were made hydrophilic by an intercalation process with CTAB, as described in the main article. The concentration of the colloidal dispersion was 3.4 mg·mL^{-1}. The presence of CTAB molecules was verified by FT-IR, as shown as an example in **Figure 12S**.

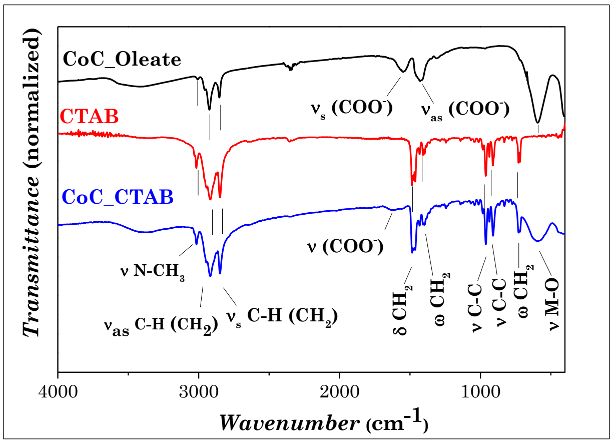

Figure 12S. FT-IR spectra of CTAB, CoC_CTAB, and CoC_Oleate samples.

The FT-IR spectrum of CoC after the intercalation process (CoC_CTAB), shows the main vibration M-O mode at 594 cm^{-1}, the bands at 2945, 2917, 2870, and 2850 cm^{-1} associated to the different modes of the hydrocarbon chain (present in both oleate and CTAB), and the bands typical of CTAB in the region 1500-500 cm^{-1} besides the peak at 3015 cm^{-1} related to the N-CH$_3$ mode. The band confirms the presence of the oleic acid molecules bonded to the nanoparticle surface at around 1550 cm^{-1}, associated with the carboxylate stretching mode.[28]

MAGNETOMETRY AND CALORIMETRIC MEASUREMENTS ON HYDROPHILIC FERROFLUIDS

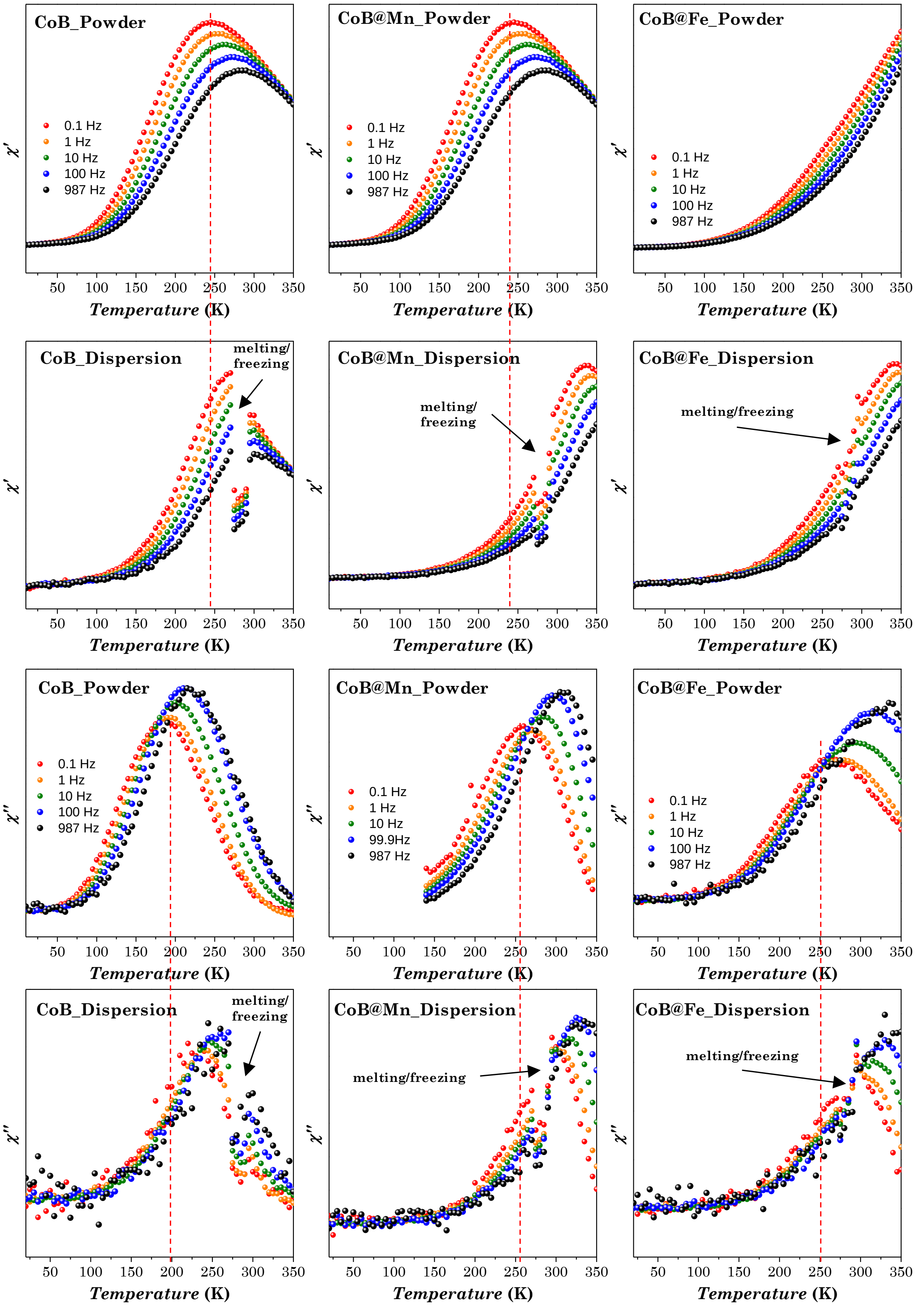

Figure 13S AC magnetic measurements on the hydrophilic ferrofluids and powdered samples of the CoB series.

To the best of our knowledge, in the literature the role of dipolar interactions in bimagnetic spinel ferrite-base core-shell nanoparticles in the heat release is not studied, due to the intrinsic complex nature of the system.[29,30,39–45,31–38] Indeed, to investigate the role of dipolar interactions it would be necessary to have no changes in composition, size, morphology, *etc.* of the primary nanoparticles, but only differences in the aggregates/agglomerates in terms of size (number of primary NPs) and shape (random or controlled clustering such as chain-like alignment). In our work, the samples differ for several features (core size, shell thickness, and chemical composition) and therefore it is not possible to conclude about the specific role of the dipolar interactions in the heat release.
The studies present in the literature about the dipolar interactions and their role in the heating dissipation are mainly devoted to single-phase nanoparticles, but their role is still debated with contradictory results showing improvement[30,34–36,41] or deterioration[30–33,45] of the heating abilities that consequently are hardly predictable. Indeed, the shape and the size of the aggregate influence the role of the dipolar interaction, being commonly beneficial when ordered clusters (*e.g.* chains) are formed, but detrimental if NPs are randomly oriented. Some authors define the dipolar coupling constant λ as:

$$\lambda = \frac{\mu_0 \mu^2}{2\pi d^3 k_B T} \quad \textbf{Eq. 20S}$$

Where μ is the magnetic moment and *d* the mean diameter. For λ>2, the system is considered strongly interacting and aggregate may happen. When λ<2, the interparticle interactions are negligible.[37] For our samples (in the form of powder), λ is always <2, therefore dipolar interaction should be negligible. Nevertheless, as we report in Figure 14S, it is evident from the FC curve that dipolar interactions are present both in the powder and in the dispersions. However, in the selected concentration range (0.7-3.4 mg/mL) no significant effect can be detected, as can be seen after rescaling the curves to relative values. We can see that both the ZFC and FC curves are almost overlapped underlying that no changes occur in the strength of dipolar interactions increasing the concentration.

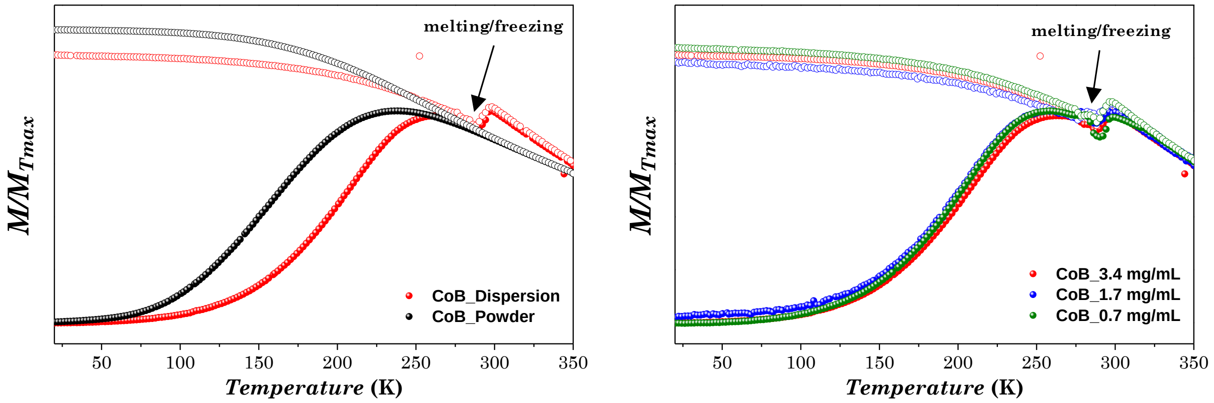

Figure 14S ZFC-FC curves of the hydrophilic ferrofluid and the powder of CoB samples (a) and ZFC-FC curves of the hydrophilic ferrofluid at different concentrations (b).

To clarify the behavior of dipolar interaction with concentration, a cobalt ferrite sample has been measured (D_{XRD} = 7.7 nm, a repeatability of the sample CoC) where both the effects of the concentration (3.4 mg/mL and 7.8 mg/mL) and the coating (cetyl trimethylammonium bromide (CTAB) and polyethylene glycol trialkoxysilane (PEG-TMS)), on SAR were studied (Figure 15S).

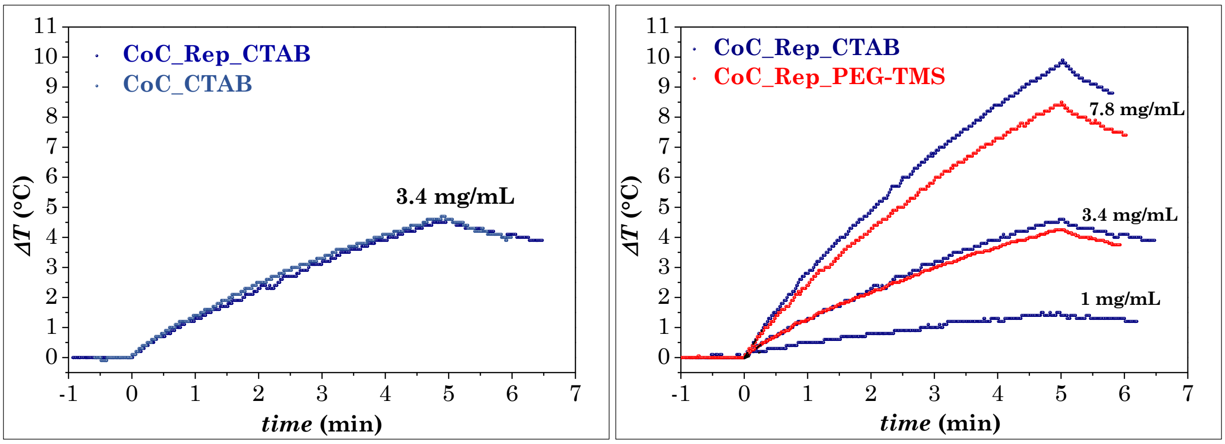

Figure 15S. Heating curves obtained on aqueous colloidal dispersions of PEG-TMS coated and CTAB coated cobalt ferrite samples at various concentrations. All curves are obtained at an initial temperature of 30 °C and under a magnetic field of f=183 kHz and H_0 = 17 kA/m.

As can be seen, even though the final temperature of the curves is different, the SAR values are equal within the experimental error at different concentrations and different coating molecules (except for the curve measured at 1 mg/mL where it is not possible to calculate the derivative *dT/dt*) and therefore they are independent of interparticle interactions.

Table 6S. SAR values normalized for the oxide phase of the water dispersion of CTAB and PEGTMS CoC and CoC_Rep samples.

Sample	D_{XRD} (nm)	Concentration (mg/mL)	Coating molecule	SAR (W/g_{ox})
CoC	8.0	3.4	CTAB	32 (2)
$CoFe_2O_4$	7.7	7.8	CTAB	30 (1)
$CoFe_2O_4$	7.7	3.4	CTAB	31 (1)
$CoFe_2O_4$	7.7	1	CTAB	-
$CoFe_2O_4$	7.7	7.8	PEG-TMS	32 (2)
$CoFe_2O_4$	7.7	3.4	PEG-TMS	32 (2)

LITERATURE MFH STUDIES OF BIMAGNETIC SPINEL FERRITE-BASED CORE-SHELL NANOPARTICLES

Table 7S. Size of the core (D_{CORE}) and of the entire core-shell NP (D_{NP}), effective anisotropy constant (K_{eff}), saturation magnetization at room temperature (M_s^{RT}), coercive field at low temperature (H_c^{LT}) or at room temperature (*) for blocked NPs, frequency (f) and amplitude (H_0) of the applied alternate magnetic field, specific adsorption rate (SAR), and intrinsic loss power (ILP).   .

System	D_{CORE} (nm)	D_{NP} (nm)	K_{eff} (J/m^3)·10^4	M_s^{RT} (Am^2/kg)	H_c^{LT} (T)	f (kHz)	H_0 (kA/m)	SAR (w/g)	ILP (nH·m^{2}kg^1)	#
$CoFe_2O_4@MnFe_2O_4$	$5.9^{\#}$	$9.7^{\#}$	1.2	75	0.92	183	17	20	0.38	This work
$CoFe_2O_4@MnFe_2O_4$	$7.5^{\#}$	$13.2^{\#}$	6.8	75	0.81			27	0.52	
$CoFe_2O_4@MnFe_2O_4$	$9.0^{\#}$	$13.3^{\#}$	4.8	70	0.56			43	0.81	
$CoFe_2O_4@MnFe_2O_4$	$9.0^{\#}$	$14.9^{\#}$	4.2	71	0.60			47	0.89	
$CoFe_2O_4@Fe_3O_4$	$5.9^{\#}$	$10.9^{\#}$	2.8	83	0.76			42	0.80	
$CoFe_2O_4@Fe_3O_4$	$7.5^{\#}$	$12.8^{\#}$	6.3	81	1.02			48	0.92	
$CoFe_2O_4@Fe_3O_4$	$9.0^{\#}$	$11.7^{\#}$	2.3	77	0.60			46	0.88	
$CoFe_2O_4@Fe_3O_4$	$9.0^{\#}$	$12.8^{\#}$	3.3	79	0.83			59	1.12	
$CoFe_2O_4@MnFe_2O_4$	$9^{\#}$	$15^{\#}$	1.5	-	0.25	500	37.3	2280	3.28	46
$CoFe_2O_4@Fe_3O_4$	$9^{\#}$	$15^{\#}$	2	-	~0.25			1120	1.60	
$MnFe_2O_4@CoFe_2O_4$	$9^{\#}$	$15^{\#}$	1.7	-	~0.8			3034	4.36	
$Fe_3O_4@CoFe_2O_4$	$9^{\#}$	$15^{\#}$	1.8	-	~1			2795	4.02	
$Zn_{0.4}Co_{0.6}Fe_2O_4@Zn_{0.4}Mn_{0.6}Fe_2O_4$	$9^{\#}$	$15^{\#}$	-	-	-			3886	5.59	
γ-$Fe_2O_3@CoFe_2O_4$	$3.9^{\S}$	$4.3^{\S}$	-	50	0.66	300	7.7	5.1	0.29	47
γ-$Fe_2O_3@CoFe_2O_4$	$3.9^{\S}$	$7.1^{\S}$	-	30	0.63			3.3	0.19	
γ-$Fe_2O_3@CoFe_2O_4$	$3.9^{\S}$	$10^{\S}$	-	-	-			1.3	0.07	
$CoFe_2O_4@MnFe_2O_4$	$6.5^{\#}$	$12.2^{\#}$	-	60	-	200	60	139	0.19	48
$MnFe_2O_4@Fe_3O_4$	$8.7^{\#}$	$11.9^{\#}$	-	60	0.2	765	23.9	40	0.09	49
$CoFe_2O_4@Fe_3O_4$	$7.2^{\#}$	$12.2^{\#}$	-	73	1.1			450	1.03	
$Zn_{0.4}Co_{0.6}Fe_2O_4@Zn_{0.4}Mn_{0.6}Fe_2O_4$	$7.6^{\#}$	$11^{\#}$	-	69	-	200	60.6	1343	1.83	50
$Zn_{0.2}Co_{0.8}Fe_2O_4@MnFe_2O_4$	-	$9.3^{\S}$	-	54	-	1955	-	380	-	51
$Zn_{0.4}Co_{0.6}Fe_2O_4@MnFe_2O_4$	-	$10.1^{\S}$	-	62	-			420	-	
$Zn_{0.6}Co_{0.4}Fe_2O_4@MnFe_2O_4$	-	$11^{\S}$	-	52	-			470	-	
$Zn_{0.8}Co_{0.2}Fe_2O_4@MnFe_2O_4$	-	$12^{\S}$	-	47	-			520	-	
$Zn_{0.17}Mn_{0.68}Fe_{1.90}O_4@$ γ-Fe_2O_3	-	$9.2^{\S}$	0.9	50	0.03	265	12	130	3.41	52
$Zn_{0.23}Mn_{0.61}Fe_{1.93}O_4@$ γ-Fe_2O_3	-	$8.8^{\S}$	1.2	48	0.03			~40	~1.05	
$Zn_{0.31}Mn_{0.52}Fe_{1.95}O_4@$ γ-Fe_2O_3	-	$6.2^{\S}$	-	47	-			~25	~0.66	
$Zn_{0.31}Mn_{0.47}Fe_{2.01}O_4@$ γ-Fe_2O_3	-	$7.4^{\S}$	-	40	-			~20	~0.52	
$Zn_{0.42}Mn_{0.37}Fe_{2.04}O_4@$ γ-Fe_2O_3	-	$5.9^{\S}$	1.5	39	0.03			~20	~0.52	
$Zn_{0.39}Mn_{0.30}Fe_{2.13}O_4@$ γ-Fe_2O_3	-	$4.5^{\S}$	-	39	-			~0	~0	
$Zn_{0.64}Mn_{0.18}Fe_{2.07}O_4@$ γ-Fe_2O_3	-	$4.9^{\S}$	-	35	-			~0	~0	
$Zn_{0.62}Mn_{0.10}Fe_{2.16}O_4@$ γ-Fe_2O_3	-	$5.3^{\S}$	1.4	32	0.03			~5	~0.13	
$Zn_{0.11}Co_{0.90}Fe_{1.99}O_4@$ γ-Fe_2O_3	-	$7.9^{\S}$	25	45	0.68			~30	~0.79	
$Zn_{0.20}Co_{0.81}Fe_{1.99}O_4@$ γ-Fe_2O_3	-	$7.5^{\S}$	-	28	-			~20	~0.52	
$Zn_{0.30}Co_{0.67}Fe_{2.02}O_4@$ γ-Fe_2O_3	-	$7.5^{\S}$	23	44	0.54			~60	~1.57	
$Zn_{0.37}Co_{0.58}Fe_{2.03}O_4@$ γ-Fe_2O_3	-	$7.9^{\S}$	-	48	-			~55	~1.44	
$Zn_{0.46}Co_{0.48}Fe_{2.04}O_4@$ γ-Fe_2O_3	-	$6.1^{\S}$	17	31	0.39			~25	~0.66	
$Zn_{0.50}Co_{0.37}Fe_{2.09}O_4@$ γ-Fe_2O_3	-	$7.9^{\S}$	-	26	-			~25	~0.66	
$Zn_{0.55}Co_{0.29}Fe_{2.11}O_4@$ γ-Fe_2O_3	-	$5.5^{\S}$	11	35	0.30			~20	~0.52	
$Zn_{0.57}Co_{0.20}Fe_{2.15}O_4@$ γ-Fe_2O_3	-	$5.5^{\S}$	-	26	-			~10	~0.26	
$Zn_{0.56}Co_{0.10}Fe_{2.23}O_4@$ γ-Fe_2O_3	-	$3.9^{\S}$	5.3	28	0.14			~20	~0.52	
$CoFe_2O_4@MnFe_2O_4$	$6.7^{\#}$	$12.9^{\#}$	3.9	-	0.5	412.5	22.4	553	2.67	53
$MnFe_2O_4@CoFe_2O_4$	$10.2^{\#}$	$14.3^{\#}$	5.3	-	1.3			302	1.46	
$MnFe_2O_4@CoFe_2O_4$	$7.3^{\#}$	$13.8^{\#}$	8.5	-	2.2			291	1.41	
$Fe_3O_4@CoFe_2O_4$	$6.4^{\S}$	$8.2^{\S}$	-	67	-	310	63.7	208	0.17	54
$Fe_3O_4@CoFe_2O_4$	$7.8^{\S}$	$9.6^{\S}$	-	68	-			199	0.16	

Fe_3O_4@$CoFe_2O_4$	7.5[#]	9.9[#]	6.6	-	~0.7	817	15.9	~120	0.58	55
Fe_3O_4@$Zn_{0.25}Co_{0.75}Fe_2O_4$	7.5[#]	10.0[#]	6.3	-	~0.5			~130	0.63	
Fe_3O_4@$Zn_{0.50}Co_{0.05}Fe_2O_4$	7.5[#]	9.4[#]	6.0	-	~0.4			~190	0.92	
Fe_3O_4@$Zn_{0.75}Co_{0.25}Fe_2O_4$	7.5[#]	9.4[#]	3.2	-	~0.2			~10	0.05	
Fe_3O_4@$ZnFe_2O_4$	7.5[#]	9.7[#]	07	-	~0			~15	0.07	
$CoFe_2O_4$@$AlFe_2O_4$	10.3[§]	14.1[§]	-	52	-	112	19.9	-	-	56
Fe_3O_4@$CoFe_2O_4$	9.1[#]	11.9[#]	-	73	1.1	765	23.9	-	-	57
Fe_3O_4@$CoFe_2O_4$	6.3[#]	6.4[#]	-	52	0.27	300	7.96	5	0.26	58
Fe_3O_4@$CoFe_2O_4$	6.3[#]	8.3[#]	-	~40	0.68			14	0.74	
Fe_3O_4@$CoFe_2O_4$	6.3[#]	11.3[#]	-	28	1.01			2	0.11	
$Zn_{0.4}Fe_{2.6}O_4$@$CoFe_2O_4$	50[#]	70[#]	-	140	0.19*	500	37.7	10600	14.92	59
$CoFe_2O_4$@$MnFe_2O_4$	-	14[§]	-	79	0.02*	50	19.9	-	-	60
$CoFe_2O_4$@$MnFe_2O_4$	15[#]	26[#]	-	33	0.17*	765	28	73	0.12	61
$MnFe_2O_4$@$CoFe_2O_4$	10[#]	16[#]	-	26	0.28*			160	0.27	
$CoFe_2O_4$@$MnFe_2O_4$	-	8.2[§]	-	45	0.2*	1955	-	300	-	51
Fe_3O_4@$CoFe_2O_4$	7.8[§]	12[§]	-	83	0.01*	310	63.7	461	0.37	54
Fe_3O_4@ $CoFe_2O_4$	7.8[§]	16.6[§]	-	68	0.05*			129	0.10	
Fe_3O_4@$CoFe_2O_4$	9.6[§]	12.4[§]	-	76	0.03*			174	0.14	

***Measured at room temperature. [#]$\langle D_{TEM} \rangle$. [§]$\langle D_{XRD} \rangle$.**

EXPERIMENTAL SYNTHESIS CONDITIONS

Table 8S Synthesis conditions for different metal oleates.

Metals Oleate	Inorganic salts	Oleic acid	NaOH	Ethanol	Water	Hexane
Co^{II}-Fe^{III}	$Fe(NO_3)_3$: 16 mmol	65 mmol	66 mmol	20 mL	20 mL	20 mL
	$Co(NO_3)_2$: 8 mmol					
Mn^{II}-Fe^{III}	$Fe(NO_3)_3$: 16 mmol	65 mmol	66 mmol	20 mL	20 mL	20 mL
	$Mn(NO_3)_2$: 8 mmol					
Fe^{II}	$FeCl_2$: 24 mmol	60 mmol	54 mmol	20 mL	20 mL	20 mL

Table 9S Synthesis condition for the pure spinel ferrite nanoparticles.

Sample	n Oleate (mmol)[a]	1-pentanol (mL)	Octanol (mL)	Toluene (mL)	Distilled water (mL)	Temperature	Reaction time
CoA	6	20	-	-	10	180 °C	10h
CoB	6	10	10	-	5	220 °C	10h
CoC	6	10	10	-	5	220 °C	10h
Co_R	3	10	10	-	5	220 °C	10h
Mn_R	6	10	-	10	5	220 °C	10h

[a]referred to M^{II}-Fe^{III} oleates (M^{II} = Co^{2+} or Mn^{2+})

Table 10S Synthesis condition for the core-shell nanoparticles.

Sample	Seeds (mg)	n Oleate (mmol)[a]	1-pentanol (mL)	Toluene (mL)	Distilled water (mL)	Temperature	Reaction time
CoA@Mn	105	1.34	10	10	5	220 °C	10h
CoA@Fe	50	2.5	10	10	5	220 °C	10h
CoB@Mn	50	1	10	10	5	220°C	10h
CoB@Fe	50	1	10	10	5	220°C	10h
CoC@Mn1	50	2.5	10	10	5	220°C	10h
CoC@Mn2	50	1.5	10	10	5	220 °C	10h
CoC@Fe1	50	4	10	10	5	220°C	10h
CoC@Fe2	50	2	10	10	5	220 °C	10h

[a]Referred to Mn^{II}-Fe^{III} Oleates (CoA@Mn; CoB@Mn; CoC@Mn1; CoC@Mn2) or Fe^{II} Oleate (CoA@Fe; CoB@Fe; CoC@Fe1; CoC@Fe2)

REFERENCES

1 M. Sanna Angotzi, A. Musinu, V. Mameli, A. Ardu, C. Cara, D. Niznansky, H. L. Xin and C. Cannas, *ACS Nano*, 2017, **11**, 7889–7900.

2 J.-M. Greneche, *Hyperfine Interact.*, 2003, **148/149**, 79–89.

3 D. Peddis, N. Yaacoub, M. Ferretti, A. Martinelli, G. Piccaluga, A. Musinu, C. Cannas, G. Navarra, J. M. Greneche and D. Fiorani, *J. Phys. Condens. Matter*, 2011, **23**, 426004.

4 E. Fantechi, G. Campo, D. Carta, A. Corrias, C. de Julián Fernández, D. Gatteschi, C. Innocenti, F. Pineider, F. Rugi and C. Sangregorio, *J. Phys. Chem. C*, 2012, **116**, 8261–8270.

5 D. Carta, M. F. Casula, A. Falqui, D. Loche, G. Mountjoy, C. Sangregorio and A. Corrias, *J. Phys. Chem. C*, 2009, **113**, 8606–8615.

6 V. Blanco-Gutiérrez, J. A. Gallastegui, P. Bonville, M. J. Torralvo-Fernández and R. Sáez-Puche, *J. Phys. Chem. C*, 2012, **116**, 24331–24339.

7 D. Carta, A. Corrias, A. Falqui, R. Brescia, E. Fantechi, F. Pineider and C. Sangregorio, *J. Phys. Chem. C*, 2013, **117**, 9496–9506.

8 V. Mameli, A. Musinu, A. Ardu, G. Ennas, D. Peddis, D. Niznansky, C. Sangregorio, C. Innocenti, N. T. K. Thanh and C. Cannas, *Nanoscale*, 2016, **8**, 10124–10137.

9 A. Repko, J. Vejpravová, T. Vacková, D. Zákutná and D. Nižňanský, *J. Magn. Magn. Mater.*, 2015, **390**, 142–151.

10 J. M. D. Coey, *Magnetism and magnetic materials*, Cambridge University Press, Cambridge, 2009.

11 K. Vamvakidis, M. Katsikini, D. Sakellari, E. C. Paloura, O. Kalogirou and C. Dendrinou-Samara, *Dalton Trans.*, 2014, **43**, 12754–65.

12 D. Peddis, D. Rinaldi, G. Ennas, A. Scano, E. Agostinelli and D. Fiorani, *Phys. Chem. Chem. Phys.*, 2012, **14**, 3162–9.

13 D. Peddis, C. Cannas, a Musinu, a Ardu, F. Orrù, D. Fiorani, S. Laureti, D. Rinaldi, G. Muscas, G. Concas and G. Piccaluga, *Chem. Mater.*, 2013, **25**, 2–10.

14 E. C. Stoner and E. P. Wohlfarth, *Philos. Trans. R. Soc. A Math. Phys. Eng. Sci.*, 1948, **240**, 599–642.

15 A. Hutlova, D. Niznansky, J.-L. Rehspringer, C. Estournès and M. Kurmoo, *Adv. Mater.*, 2003, **15**, 1622–1625.

16 A. Repko, D. Nižňanský and J. Poltierová-Vejpravová, *J. Nanoparticle Res.*, 2011, **13**, 5021–5031.

17 D. Peddis, F. Orrù, A. Ardu, C. Cannas, A. Musinu and G. Piccaluga, *Chem. Mater.*, 2012, **24**, 1062–1071.

18 J. Vejpravova, V. Sechovsky, J. Plocek, D. Niznansky, A. Hutlova and J. L. Rehspringer, *J. Appl. Phys.*

19 A. López-Ortega, M. Estrader, G. Salazar-Alvarez, A. G. Roca and J. Nogués, *Phys. Rep.*, 2015, **553**, 1–32.

20 R. W. Chantrell, M. El-Hilo and K. O'Grady, *IEEE Trans. Magn.*, 1991, **27**, 3570–3578.

21 S. Bedanta and W. Kleemann, *J. Phys. D. Appl. Phys.*, 2009, **42**, 013001.

22 D. Peddis, M. Hudl, C. Binns, D. Fiorani and P. Nordblad, *J. Phys. Conf. Ser.*, 2010, **200**, 072074.

23 G. Margaris, M. Vasilakaki, D. Peddis, K. N. Trohidou, S. Laureti, C. Binns, E. Agostinelli, D. Rinaldi, R. Mathieu and D. Fiorani, *Nanotechnology*, 2017, **28**, 035701.

24 J. Van Rijssel, B. W. M. Kuipers and B. H. Erné, *J. Magn. Magn. Mater.*, 2014, **353**, 110–115.

25 A. Boni, A. M. Basini, L. Capolupo, C. Innocenti, M. Corti, M. Cobianchi, F. Orsini, A. Guerrini, C. Sangregorio and A. Lascialfari, *RSC Adv.*, 2017, **7**, 44104–44111.

26 G. Muscas, N. Yaacoub, G. Concas, F. Sayed, R. Sayed Hassan, J. M. Greneche, C. Cannas, A. Musinu, V. Foglietti, S. Casciardi, C. Sangregorio and D. Peddis, *Nanoscale*, 2015, **7**, 13576–13585.

27 E. P. Shtrikman, S and Wohlfarth, *Phys. Lett.*, 1981, **85**, 467–470.

28 N. Shukla, C. Liu, P. M. Jones and D. Weller, *J. Magn. Magn. Mater.*, 2003, **266**, 178–184.

29 R. D. Ralandinliu Kahmei and J. P. Borah, *Nanotechnology*, 2019, **30**, 035706.

30 A. F. Abu-Bakr and A. Y. Zubarev, *J. Magn. Magn. Mater.*, 2019, **477**, 404–407.

31 C. Haase and U. Nowak, *Phys. Rev. B - Condens. Matter Mater. Phys.*, 2012, **85**, 2–6.

32 L. C. Branquinho, M. S. Carrião, A. S. Costa, N. Zufelato, M. H. Sousa, R. Miotto, R. Ivkov and A. F. Bakuzis, *Sci. Rep.*, 2013, **3**, 20–22.

33 C. Blanco-Andujar, D. Ortega, P. Southern, Q. A. Pankhurst and N. T. K. Thanh, *Nanoscale*, 2015, **7**, 1768–1775.

34 C. L. Dennis, A. J. Jackson, J. A. Borchers, R. Ivkov, A. R. Foreman, J. W. Lau, E. Goernitz and C. Gruettner, *J. Appl. Phys.*, 2008, **103**, 2–5.

35 A. Y. Zubarev, *Phys. Rev. E*, , DOI:10.1103/PhysRevE.98.032610.
36 B. Mehdaoui, R. P. Tan, A. Meffre, J. Carrey, S. Lachaize, B. Chaudret and M. Respaud, *Phys. Rev. B - Condens. Matter Mater. Phys.*, 2013, **87**, 1–10.
37 Y. Hadadian, A. P. Ramos and T. Z. Pavan, *Sci. Rep.*, 2019, **9**, 18048.
38 L. L. Gutiérrez, L. de la Cueva, M. Moros, E. Mazarío, S. de Bernardo, J. M. de la Fuente, M. P. Morales and G. Salas, *Nanotechnology*, 2019, **30**, 112001.
39 D. F. Coral, P. Mendoza Zélis, M. Marciello, M. D. P. Morales, A. Craievich, F. H. Sánchez and M. B. Fernández Van Raap, *Langmuir*, 2016, **32**, 1201–1213.
40 C. Martinez-Boubeta, K. Simeonidis, A. Makridis, M. Angelakeris, O. Iglesias, P. Guardia, A. Cabot, L. Yedra, S. Estradé, F. Peiró, Z. Saghi, P. A. Midgley, I. Conde-Leborán, D. Serantes and D. Baldomir, *Sci. Rep.*, 2013, **3**, 1–8.
41 D. Serantes, K. Simeonidis, M. Angelakeris, O. Chubykalo-Fesenko, M. Marciello, M. Del Puerto Morales, D. Baldomir and C. Martinez-Boubeta, *J. Phys. Chem. C*, 2014, **118**, 5927–5934.
42 P. De La Presa, Y. Luengo, V. Velasco, M. P. Morales, M. Iglesias, S. Veintemillas-Verdaguer, P. Crespo and A. Hernando, *J. Phys. Chem. C*, 2015, **119**, 11022–11030.
43 C. Guibert, V. Dupuis, V. Peyre and J. Fresnais, *J. Phys. Chem. C*, 2015, **119**, 28148–28154.
44 D. Niculaes, A. Lak, G. C. Anyfantis, S. Marras, O. Laslett, S. K. Avugadda, M. Cassani, D. Serantes, O. Hovorka, R. Chantrell and T. Pellegrino, *ACS Nano*, 2017, **11**, 12121–12133.
45 B. Mehdaoui, A. Meffre, J. Carrey, S. Lachaize, L.-M. Lacroix, M. Gougeon, B. Chaudret and M. Respaud, *Adv. Funct. Mater.*, 2011, **21**, 4573–4581.
46 J.-H. Lee, J.-T. Jang, J.-S. Choi, S. H. Moon, S.-H. Noh, J.-G. J.-W. J.-G. J.-W. Kim, J.-G. J.-W. J.-G. J.-W. Kim, I.-S. Kim, K. I. Park and J. Cheon, *Nat. Nanotechnol.*, 2011, **6**, 418–422.
47 O. V. Yelenich, S. O. Solopan, J. M. Greneche and A. G. Belous, *Solid State Sci.*, 2015, **46**, 19–26.
48 J. Wang, Z. Zhou, L. Wang, J. Wei, H. Yang, S. Yang and J. Zhao, *RSC Adv.*, 2015, **5**, 7349–7355.
49 S. Liébana-Viñas, K. Simeonidis, U. Wiedwald, Z.-A. Li, Z. Ma, E. Myrovali, A. Makridis, D. Sakellari, G. Vourlias, M. Spasova, M. Farle and M. Angelakeris, *RSC Adv.*, 2016, **6**, 72918–72925.
50 L. Wang, Y. Yan, M. Wang, H. Yang, Z. Zhou, C. Peng and S. Yang, *J. Mater. Chem. B*, 2016, **4**, 1908–1914.
51 M. Hammad, V. Nica and R. Hempelmann, *IEEE Trans. Magn.*, 2017, **53**, 1–6.
52 V. Pilati, R. Cabreira Gomes, G. Gomide, P. Coppola, F. G. Silva, F. L. O. Paula, R. Perzynski, G. F. Goya, R. Aquino and J. Depeyrot, *J. Phys. Chem. C*, 2018, **122**, 3028–3038.
53 Q. Zhang, I. Castellanos-Rubio, R. Munshi, I. Orue, B. Pelaz, K. I. Gries, W. J. Parak, P. Del Pino and A. Pralle, *Chem. Mater.*, 2015, **27**, 7380–7387.
54 J. Robles, R. Das, M. Glassell, M. H. Phan and H. Srikanth, *AIP Adv.*, 2018, **8**, 2–8.
55 F. Fabris, E. Lima, E. De Biasi, H. E. Troiani, M. Vásquez Mansilla, T. E. Torres, R. Fernández Pacheco, M. R. Ibarra, G. F. Goya, R. D. Zysler and E. L. Winkler, *Nanoscale*, 2019, **11**, 3164–3172.
56 H. Choi, M. An, W. Eom, S. W. Lim, I. B. Shim, C. S. Kim and S. J. Kim, *J. Korean Phys. Soc.*, 2017, **70**, 173–176.
57 S. L. Viñas, K. Simeonidis, Z. A. Li, Z. Ma, E. Myrovali, A. Makridis, D. Sakellari, M. Angelakeris, U. Wiedwald, M. Spasova and M. Farle, *J. Magn. Magn. Mater.*, 2016, **415**, 20–23.
58 S. O. Solopan, N. Nedelko, S. Lewińska, A. Ślawska-Waniewska, V. O. Zamorskyi, A. I. Tovstolytkin and A. G. Belous, *J. Alloys Compd.*, 2019, **788**, 1203–1210.
59 S. Noh, W. Na, J. Jang, J.-H. Lee, E. J. Lee, S. H. Moon, Y. Lim, J.-S. Shin and J. Cheon, *Nano Lett.*, 2012, **12**, 3716–3721.
60 M. S. Kim, C. S. Kim, H. J. Kim, K.-H. H. Yoo and E. J. Hahn, *J. Korean Phys. Soc.*, 2013, **63**, 2175–2178.
61 M. Angelakeris, Z.-A. A. Li, M. Hilgendorff, K. Simeonidis, D. Sakellari, M. Filippousi, H. Tian, G. Van Tendeloo, M. Spasova, M. Acet and M. Farle, *J. Magn. Magn. Mater.*, 2015, **381**, 179–187.
